# De novo design of peptides that coassemble into β sheet–based nanofibrils

**DOI:** 10.1126/sciadv.abf7668

**Published:** 2021-09-03

**Authors:** Xingqing Xiao, Yiming Wang, Dillon T. Seroski, Kong M. Wong, Renjie Liu, Anant K. Paravastu, Gregory A. Hudalla, Carol K. Hall

**Affiliations:** 1 Department of Chemical and Biomolecular Engineering, North Carolina State University, Raleigh, NC 27695-7905, USA.; 2 Department of Biomedical Engineering, University of Florida, Gainesville, FL 32611, USA.; 3 Department of Chemical and Biomolecular Engineering, Georgia Institute of Technology, Atlanta, GA 30332, USA.

## Abstract

Peptides’ hierarchical coassembly into nanostructures enables controllable fabrication of multicomponent biomaterials. In this work, we describe a computational and experimental approach to design pairs of charge-complementary peptides that selectively coassemble into β-sheet nanofibers when mixed together but remain unassembled when isolated separately. The key advance is a peptide coassembly design (PepCAD) algorithm that searches for pairs of coassembling peptides. Six peptide pairs are identified from a pool of ~10^6^ candidates via the PepCAD algorithm and then subjected to DMD/PRIME20 simulations to examine their co-/self-association kinetics. The five pairs that spontaneously aggregate in kinetic simulations selectively coassemble in biophysical experiments, with four forming β-sheet nanofibers and one forming a stable nonfibrillar aggregate. Solid-state NMR, which is applied to characterize the coassembling pairs, suggests that the in silico peptides exhibit a higher degree of structural order than the previously reported CATCH(+/−) peptides.

## Teaser

Computational discoveries and experimental characterizations of coassembly peptides that form β sheet–based nanofibrils.

## INTRODUCTION

Certain peptides are known to assemble spontaneously into a variety of nanostructures—nanofibers, nanosheets, nanotubes, nanoparticles, etc.—with applications in a wide variety of fields, including drug delivery, vaccines, hydrogels, three-dimensional (3D) cell culture, tissue engineering, and protein scaffolds. Great structural variety can be achieved, in principle, via a “bottom-up” strategy in which the peptide amino acid composition, length, and sequence pattern are tailored to form a particular structure (*1*–*7*). The big question is, of course, what are the design rules for programming in a particular self-assembled structure? An even more basic question is which sequences will assemble? Although there are some algorithms that attempt to answer the latter question by correlating the amyloidogenic tendencies of the individual amino acids (*8*–*11*), there is no efficient computational or experimental approach to discover which sequences form which structures. Most of the existing β sheet–forming peptides are derived from naturally occurring amyloid-forming proteins (*12*). Others (*13*–*15*) have been designed on the basis of a simple hydrophilic/hydrophobic (HP)*_n_* repeating pattern that is known to form a two-layer fibril with a hydrophobic core (*16*). The difficulty in a priori design of β sheet–forming peptides comes from the challenge of effectively exploring the enormous amino acid sequence space to discover peptide sequences that form the desired structures. For this reason, systematic investigation of peptide aggregation behaviors in vast sequence space has only been conducted for very short peptides, such as dipeptides (*17*) and tripeptides (*18*), and only for single-component systems. The challenge becomes even more interesting when one considers the possibility of designing two or more peptides that coassemble to form a single nanostructure.

Recently, peptide coassembly has emerged as a novel supramolecular design strategy, allowing construction of peptide-based nanofibers with integrated functionalities (*19*–*22*). Here, we focus on selective coassembly: the formation of stable β-sheet nanofibers by two different peptides, A and B, only when they are both present in solution; otherwise, they remain unassembled in random coil configurations. Researchers have used heuristics to develop pairs of charge-complementary coassembling peptides by generating highly charged sequence variants of established self-assembling peptides (*23*, *24*). For instance, the CATCH(4+) peptide (sequence: Ac-QQKFKFKFKQQ-Am) with four positively charged residues and the CATCH(6−) peptide (sequence: Ac-EQEFEFEFEQE-Am) with six negatively charged residues (*24*) are both derived from the Q11 peptide (sequence: Ac-QQKFQFKQEQQ-Am) (*25*) and have been shown to coassemble. These peptide pairs have been shown to coassemble into bilayer β sheets that contain the two peptides arranged in a predominantly alternating pattern, although like-charged neighbor mismatches and β-strand polymorphisms have been observed (*26*,*27*). Although a heuristics-based experimental approach has led to the discovery of several coassembling pairs, this approach becomes intractable when exploring the vast sequence space for two complementary 11-residue peptides. The addition of computational methodologies capable of designing new selectively coassembling pairs would greatly accelerate the development of peptide nanostructures, potentially leading to architectures with more precise molecular-level order and organization than has heretofore been possible.

**Fig. 1.**
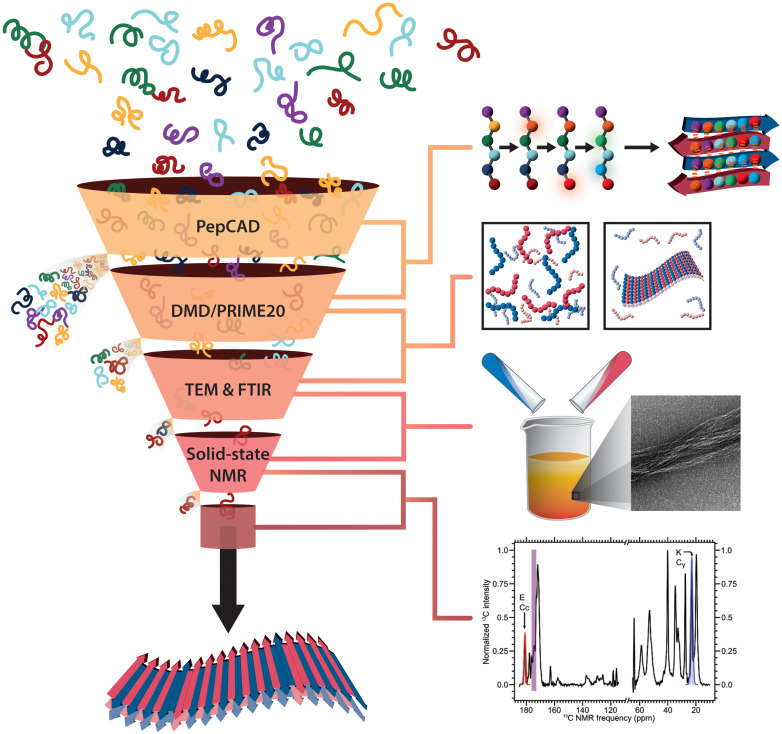
An overview of our computational and experimental protocol for identifying new pairs of peptides A and B that selectively coassemble into long-ranged β-sheet nanofibers.

In this work, we describe a computational and experimental protocol, essentially a funnel, which screens large numbers of candidate peptide pairs to identify those that will selectively coassemble into β-sheet nanofibers with a preset structure in experiment, e.g., an automated sequence screening process for 11-mer coassembling peptide pairs in a pool of more than 300,000 peptides and around 10^6^ possible peptide pairs (Fig. 1). The key advance is a Monte Carlo (MC)–type peptide coassembly design (PepCAD) algorithm that is used for de novo design of charge-complementary peptide pairs. This method is a logical extension of our previously developed peptide binding design (PepBD) algorithm (*28*–*32*) that we applied to design peptide binders to biomolecular targets with exceptional affinities (*33*–*35*). Lead compounds from the computational search are subjected to discontinuous molecular dynamics (DMD) simulations combined with the knowledge-based PRIME20 force field (*36*–*40*) to examine the coassembly kinetics of the in silico discovered peptide pairs and the self-association of each peptide species when alone. The peptide pairs that can selectively coassemble in DMD/PRIME20 simulations are then synthesized, and their coassembly versus self-association is examined using transmission electron microscopy (TEM), Fourier transform infrared spectroscopy (FTIR), and solid-state nuclear magnetic resonance (NMR) spectroscopy. Last, the structural order and molecular-level compositions are assessed by solid-state NMR and compared to previous coassembling β-sheet designs. We envision that this new paradigm for de novo peptide design will enable rapid development of molecules that assemble into specific supramolecular architectures.

## RESULTS

### Predetermining the molecular architecture of the peptide scaffold

**Fig. 2.**
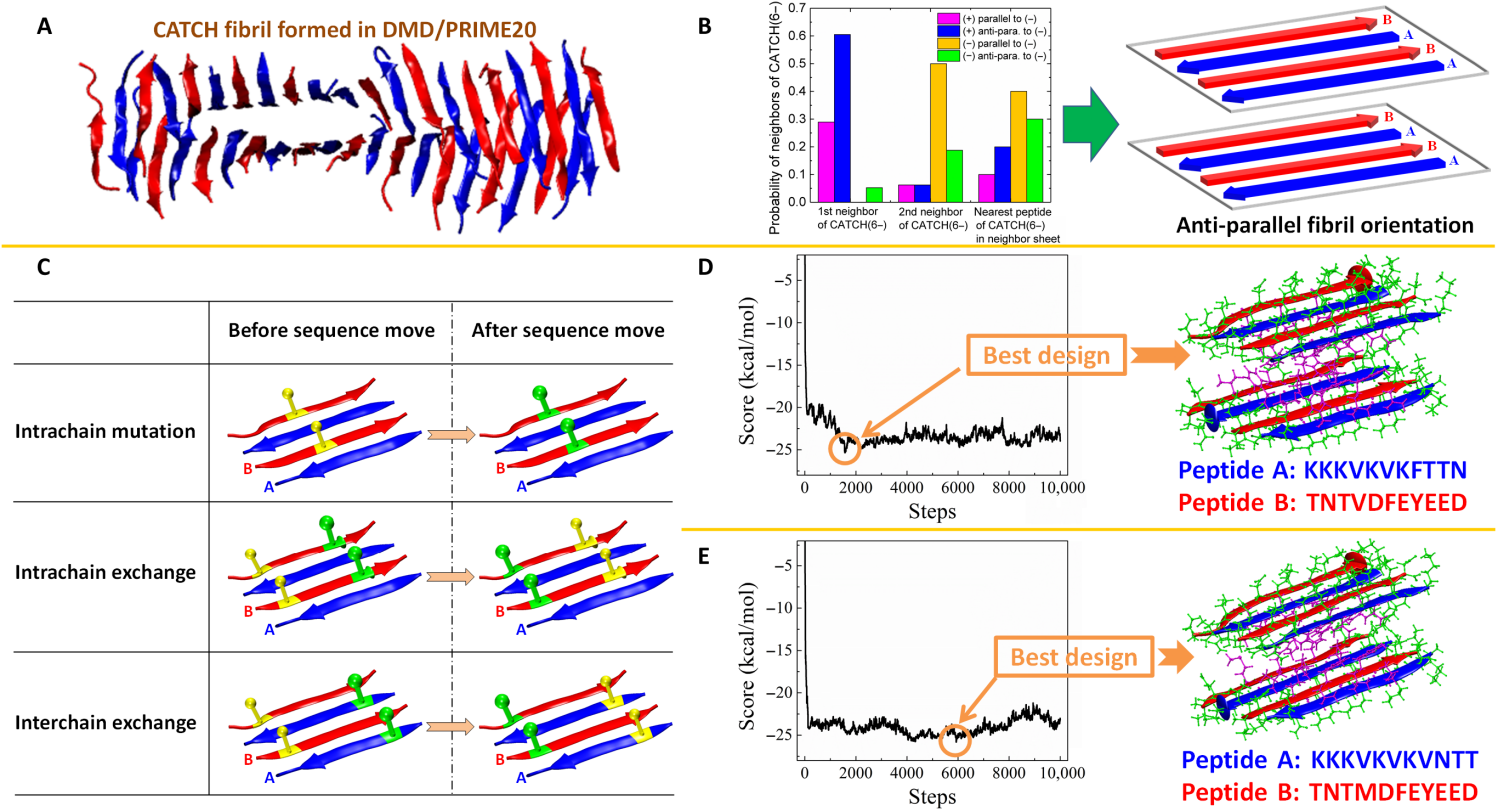
Computational modeling of CATCH peptide fibril and in silico evolution of coassembling peptides. (**A**) The DMD/PRIME20 simulation result suggests that the CATCH(4+) and CATCH(6−) peptides preferentially coassemble into a two-layer fibril structure that belongs to the eighth class of steric zippers introduced by Sawaya *et al*. (*12*). (**B**) Conformational analysis of the two-layer amyloid fibril indicates that the CATCH fibril favors having an antiparallel peptide conformation in each β sheet and that the two neighbor β sheets align parallel to each other. (**C**) Three kinds of sequence moves, viz. intrachain residue mutation, intrachain residue exchange, and interchain residue exchange, are involved in PepCAD to generate new sequences for peptides A and B. Starting from random sequences and setting (**D**) λ = 3.0 and (**E**) λ = 4.0, the algorithm searches through large numbers of possibly coassembling peptides, A and B. Plots of score versus the number of evolution steps are shown on the left. Lower scores imply better peptide designs. The best designs from the two searches are circled in the plots; their corresponding fibril structures are shown on the right.

To discover peptide pairs that can coassemble into a peptide-based nanofiber in solution, we must first decide what fibrillar structure to design—β-sheet nanofiber, cross-α nanofibers, α-helical coiled coil, etc. Here, we choose to design peptides that can assemble into the coassembled fibril structure formed by a mixture of CATCH(4+) and CATCH(6−) peptides. DMD is combined with the coarse-grained protein force field, PRIME20, to simulate the spontaneous coassembly of an initially random system of 24 CATCH(4+) peptides and 24 CATCH(6−) peptides at 330 K and 10 mM into a fibrillar structure. PRIME20 was chosen because it is among the most realistic of the protein coarse-grained models, does not build in any predetermined secondary structure, provides a good representation of amyloid structure in comparison to experiment, and is fast enough to get to the fibrillar stage starting from the random coil state (section S1). Our simulation results revealed that the CATCH(4+/6−) peptides preferentially coassemble into a highly ordered fibrillar structure with two layers of β sheets (Fig. 2A). The structure of this fibril can be characterized in terms of the orientations of the peptides relative to each other. Conformational analysis of the CATCH fibrillar structure as described in section S2 indicates that, for a CATCH(6−) peptide in the fibril, its nearest and next-nearest peptides in the same β sheet are most likely to be a CATCH(4+) peptide that is antiparallel and a CATCH(6−) peptide that is parallel, respectively, and its nearest peptide on the neighboring β sheet is most likely to be a CATCH(6−) peptide that is parallel (Fig. 2B). This preferred organization indicates that the CATCH(4+/6−) peptides prefer to coaggregate into two layers with antiparallel orientation within each β sheet and parallel orientation between the sheets (Fig. 2B). On the basis of the structural information above, we constructed a two-layer fibril model using the Discovery Studio 3.5 package and optimized its geometry in the AMBER14 software (as seen in section S3 and fig. S1). This two-layer fibril structure is used as an initial conformation in the PepCAD algorithm to discover other potential coassembling peptides.

### Sequence evolution of coassembling peptides

The PepCAD algorithm is an MC-based algorithm for de novo design of charge-complementary peptide pairs that can coassemble into particular supramolecular architectures (as described in section S4 and fig. S2). Three different kinds of sequence moves, viz. intrachain residue mutation, intrachain residue exchange, and interchain residue exchange, are used to perturb the peptide sequences (Fig. 2C), resulting in new trial coassembling designs, peptides A and B. The peptide backbone scaffold of the two-layer fibril structure is fixed throughout the design process. To evaluate the merits of these peptides’ coassembly capability, a score function Γ_score_ is introduced in section S5, eq. S1, that takes into account the binding free energy, Δ*G*_binding_, between peptides A and B (*41*), as well as the intrinsic self-aggregation propensities, *P*_agg_, of the individual peptides (*42*–*45*). To avoid local searches, we start with three different initial random sequences at two weighting factors λ, viz. λ = 3.0 and 4.0, to vary the search pathways, leading to a total of six evolution runs (three for λ = 3.0 and three for λ = 4.0). The designed peptides are constrained to have the same sequence pattern “PPPHPHPHPPP” as the CATCH(+/−) peptides, where “H” and “P” refer to hydrophobic and polar amino acids, respectively. The peptides are also constrained to have three hydrophobic residues, three hydrophilic residues, five charged residues, and no cysteine, proline, or glycine. Our sequence searches did not start with the known CATCH(4+/6−) peptides because (i) the PepCADs in this work are constrained to contain (5+/5−) residues on the chains, which is different from those for the CATCH(4+/6−) peptides, and (ii) the sequence searches starting with the CATCH(4+/6−) peptides as initial conditions result in an ineffective exploration of the broad sequence space. The final peptide designs are not sensitive to the initial conditions (sequences) chosen during the MC algorithm.

Figure 2 (D and E) shows profiles of score versus number of evolution steps at λ = 3.0 and 4.0, respectively. Because the searches start from random peptide sequences, the scores are high at the initial stage. As the evolution proceeds, the three kinds of sequence moves help the amino acids to rapidly find appropriate sites on the chains, resulting in a sharp drop in the score. Later, the score profile fluctuates considerably due to variations in the identities of the amino acids at the various sites. Favorable sequence moves that decrease the score are always accepted in our procedure, while unfavorable sequence moves that may slightly or significantly increase the score are accepted or rejected according to the MC criterion. By examining the profile of the score over the sequence evolution, we can identify the lowest scores in each profile, which correspond to the best peptide sequences for each search. Two pairs of designed coassembly peptides and their corresponding fibril structures are shown in Fig. 2 (D and E) for the case of λ = 3.0 with the lowest score −25.35 kcal/mol at the 1570th step (Fig. 2D) and for the case of λ = 4.0 with the lowest score −25.80 kcal/mol at the 5926th step (Fig. 2E).

The six best-scoring pairs of peptide sequences resulting from the six evolutions, hereafter referred to as designs 1 to 6, are listed in Table 1 along with their associated scores, binding free energies per peptide, and intrinsic self-aggregation propensities per peptide calculated using eq. S1. (The latter two quantities are used in the calculation of the peptide’s score.) The results from the DMD/PRIME20 kinetics simulations and experiments are also listed and will be discussed later. All of the in silico peptide pairs A and B in Table 1 exhibit negative values for $\Delta {\tilde{G}}_{\text{binding}}$, the first term in the score function, implying that peptides A and B may form fibril-like coaggregates due to their strong mutual binding affinity. Note that these binding energies are stronger than a typical peptide-biomolecule binding energy because the two-layer fibril model used for peptide designs is an ideal optimized structure. Each peptide within the fibril model can form at least six backbone hydrogen bonds with its nearest neighboring peptides and broadly interact with the other peptides on the neighbor sheet(s), leading to a large value of binding energy. In addition, peptide pairs A and B exhibit weak intrinsic self-aggregation propensities due to their low values of ${\tilde{P}}_{\text{agg}}$, indicating that they are not likely to self-assemble into well-ordered structures when dissolved separately in solution; the lower the value of ${\tilde{P}}_{\text{agg}}$, the weaker its self-aggregation propensity. Without loss of generality, we henceforth assign label A to the positively charged peptide and label B to the negatively charged peptide. The design algorithm, not the user-defined sequence specifications, preferentially places positively charged amino acid, lysine (K), at the N terminus of peptide A and negatively charged amino acids, aspartic acid (E) and glutamic acid (D), at the C terminus of peptide B. Such sequence alignments for peptides A and B likely facilitate the formation of the targeted antiparallel β-sheet structure. The frequent appearances of the amino acids asparagine (N) and threonine (T) on peptide pairs A and B are likely due to their low intrinsic self-aggregation propensities as well as the strong side chain–side chain interactions between the carboxamide group (−CO-NH_2_) on asparagine (N) and the hydroxyl group (−OH) in threonine (T), which further stabilize the amyloid fibril. These design motifs are not seen in previous coassembling β-sheet peptide sequences, highlighting the utility of the design algorithm in expanding the design space.

**Table 1.** The sequences of the six in silico discovered peptide pairs, their associated scores (Γ_score_), binding free energies per peptide ($\Delta {\tilde{G}}_{\text{binding}}$), intrinsic self-aggregation propensities per peptide (${\tilde{P}}_{\text{agg}}$), the DMD/PRIME20 simulation results, and the TEM-observed results (unit: kcal/mol). Designs 1 to 3 result from setting λ = 3.0, while designs 4 to 6 result from setting λ = 4.0.

**Designs**		**Sequences and sites**											**Γ_score_**	** $\Delta {\tilde{G}}_{\text{binding}}$ **	** ${\tilde{P}}_{\text{agg}}$ **	**DMD/** **PRIME20**	**TEM**
		**1**	**2**	**3**	**4**	**5**	**6**	**7**	**8**	**9**	**10**	**11**					
1	**Peptide A**	K	K	K	M	K	V	K	V	N	T	T	−25.40	−25.07	−0.11	Multilayer fibril	Short nanofiber
	**Peptide B**	T	N	T	A	D	F	E	F	E	E	D					
2	**Peptide A**	K	K	K	V	K	V	K	F	T	T	N	−25.35	−24.93	−0.14	Multilayer fibril	Long nanofiber
	**Peptide B**	T	N	T	V	D	F	E	Y	E	E	D					
3	**Peptide A**	K	K	K	W	K	M	K	A	T	N	T	−26.85	−25.87	−0.33	Random coils	Not performed
	**Peptide B**	T	N	T	V	E	V	E	L	D	D	D					
4	**Peptide A**	K	K	K	V	K	V	K	V	N	T	T	−25.62	−25.16	−0.12	Multilayer fibril	Long nanofiber
	**Peptide B**	T	N	T	A	E	F	E	F	E	E	D					
5	**Peptide A**	K	K	K	V	K	V	K	V	N	T	T	−25.80	−25.62	−0.05	Multilayer fibril	Aggregated fibrils
	**Peptide B**	T	N	T	M	D	F	E	Y	E	E	D					
6	**Peptide A**	K	K	K	V	K	Y	T	F	K	N	T	−25.93	−25.21	−0.18	Long two-layer fibril	Nonfibrillar aggregates
	**Peptide B**	T	N	T	M	E	V	D	F	D	E	D					

### Computational analysis of the co-/self-assembly properties of in silico peptide pairs

First, we performed explicit-solvent atomistic molecular dynamics (MD) simulations to examine the thermodynamic stability of our designed amyloid fibrils (as described in section S6). The starting structures of the six two-layer amyloid fibrils are obtained from the output of the PepCAD algorithm. Simulation results revealed that the six in silico peptide pairs are able to maintain a well-organized two-layer fibril structure after 100 ns (fig. S3). We then used the FoldAmyloid Web server, a bioinformatics method, to estimate the amyloidogenicity (likelihood that the peptides would self-aggregate to form amyloid) of the single peptide species within the six designs (*46*). In this method, peptides are predicted to be amyloidogenic if they contain at least seven consecutive residues that have average self-aggregation scales that are higher than an empirical threshold value of 21.4; otherwise, they are predicted to be nonamyloidogenic. Peptide pairs A and B within designs 1 to 6 are predicted to be nonamyloidogenic, as their average self-aggregation scales are lower than 21.4, implying that these single peptide species exhibit a weak propensity for self-aggregation in solution (fig. S3).

**Fig. 3.**
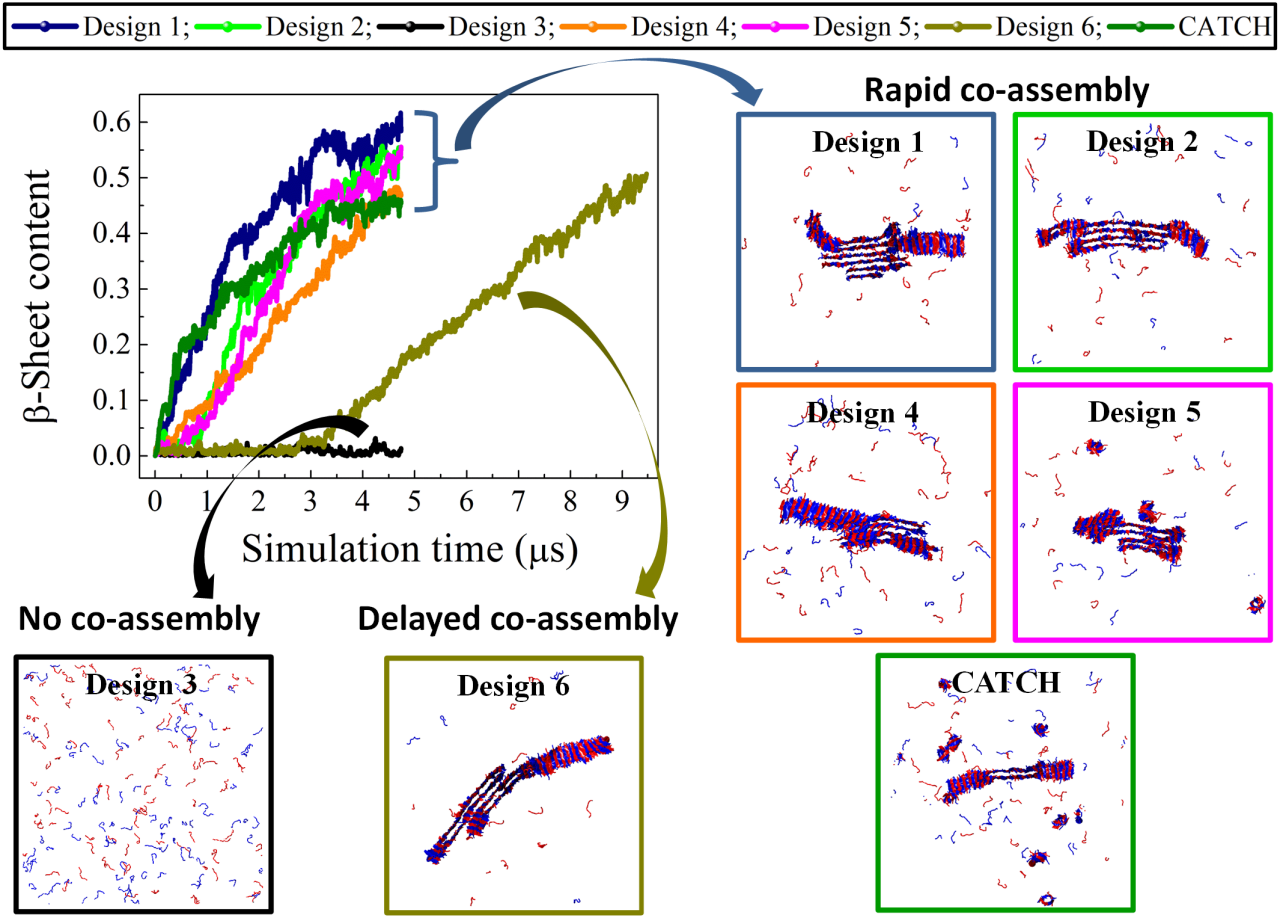
DMD/PRIME20 simulations of peptide coaggregation. Plots of β-sheet content versus simulation time describe the coaggregation kinetics for mixtures of 100A and 100B peptides in designs 1 to 6 and CATCH(+/−). Snapshots of the final simulation structures of the seven systems are shown as well.

In addition, the co- and self-association kinetics of these in silico peptide pairs were examined using DMD/PRIME20 simulations. Motivation for this is the possibility that although the fibril state might be stable according to the atomistic MD simulations, the kinetics might not be fast enough to arrive at an ordered structure. DMD/PRIME20 simulations of a mixture containing 100A and 100B peptides initially in random coil conformations at 10 mM and 330 K were conducted for 5 (or 10) μs (as described in section S7). The types of structure formed for all six designs in DMD/PRIME 20 simulations are indicated in Table 1. Figure 3 shows the aggregation kinetics, reported as the β-sheet content versus simulation time, and final simulation snapshots for designs (1 to 6) and CATCH peptides. Our simulation results predict that all the in silico peptide pairs selectively coassemble into ordered β-sheet fibrillar structures, with the exception of design 3 (Fig. 3). Peptide pairs A and B of designs (1, 2, 4, and 5) coaggregate rapidly when mixed with each other to form fibril structures with more than two β-sheet layers, wherein peptides A and B predominantly adopt an antiparallel orientation (Fig. 3). In contrast, design 6 has the slowest aggregation kinetics (Fig. 3) but is the only peptide pair that tends to form a two-layer architecture (Fig. 3), as the CATCH peptides do. The coassembly of peptides A and B within these designs is initialized by the formation of a small β-sheet fibril nucleus. As the simulation progresses, the nucleus grows and elongates by recruiting random coil monomeric peptides and, sometimes, by associating laterally with other small oligomeric species to form a single multilayer β-sheet fibrillar structure (fig. S4). The DMD/PRIME20 simulations of the associated single-component systems reveal that the individual peptides, A or B, in the six designs do not self-associate when alone in solution (fig. S5).

To study the effectiveness of PepCAD, we chose three initial random peptide pairs and three in silico peptide pairs with medium scores from the evolution for coassembly to test to see whether they coassembled in the DMD/PRIME20 simulations. Simulation results, which are shown in fig. S6, revealed that all three initial peptide pairs do not coassemble into fibril structures. However, two of the three in silico peptides with medium scores do form fibril-like coaggregates in simulations, while the other one does not. Furthermore, the individual peptides associated with these initial pairs and pairs with medium scores did not self-assemble in DMD simulations (not shown for brevity). To examine the diversity of sequences, we compared the peptide pairs with medium and best scores (Table 1 and fig. S6) and found that the best-scoring peptides A (and B) exhibit a high similarity with each other but significantly differ from those with medium scores. Sequence evolution in PepCAD achieves the de novo design of peptides that coassemble into β sheet–based nanofibrils. Future efforts will seek to identify threshold scores that are reliable predictors of coassembly propensity in simulations and experiments.

### Experimental analyses of co- and self-assembly of designed peptide pairs

**Fig. 4.**
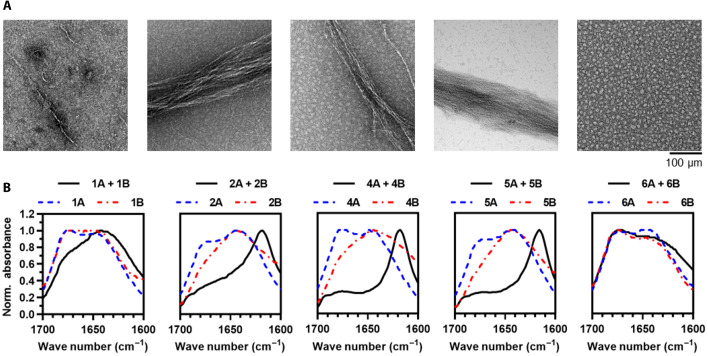
Experimental characterization of co- and self-assembly of peptide pairs in designs 1, 2, 4, 5, and 6. (**A**) Transmission electron micrographs of mixtures of designs 1, 2, 4, 5, and 6. (**B**) FTIR spectra of the peptides of designs 1, 2, 4, 5, and 6 alone (dashed lines) and in combination (solid line).

On the basis of the outcomes of the DMD/PRIME20 simulations, the peptide pairs of designs 1 to 6 were synthesized and their selective coassembly was characterized using TEM (section S8) and FTIR (section S9) (Fig. 4). We observed elongated nanofibers with a high degree of lateral association in transmission electron micrographs of equimolar mixtures of designs 2, 4, and 5 (Fig. 4A). In contrast, the mixture of design 1 formed shorter nanofibers that were few in number and less laterally aggregated (Fig. 4A). Unexpectedly, the peptide pair in design 6 did not form elongated nanofibers, contrary to simulation predictions; instead, design 6 exclusively formed nonfibrillar aggregates with approximate diameters of 11 ± 1.7 nm (Fig. 4A). Likewise, the peptide pair in design 3 formed nonfibrillar aggregates, which persisted for 7 days (fig. S8). Note that these samples were prepared at 1 mM, which is significantly lower than the simulation concentration of 10 mM, yet significantly higher than the minimum coassembly concentration reported previously for CATCH peptides.

Because of the disparity in concentration between simulations and TEM samples, we used FTIR to determine the secondary structure of each peptide in designs 1 to 6 alone and in the presence of its complementary partner at 15 mM (Fig. 4B). When alone, the FTIR spectrum of each peptide had local maxima at approximately 1645 and 1675 cm^−1^ (dashed lines). The former indicates that the peptides adopt random coil conformations and therefore do not undergo considerable self-association. The latter is likely due to residual trifluoroacetic acid remaining from peptide synthesis and purification processes, and likely has no impact on the peptide secondary structure. When paired, the FTIR spectra of designs 4 and 5 had strong maxima between 1621 and 1616 cm^−1^, indicating formation of intermolecular hydrogen bonds consistent with a β-sheet secondary structure. The design 2 FTIR spectrum had a major peak near 1620 cm^−1^ but also had significant absorption in the range of 1630 to 1690 cm^−1^, suggesting a lesser abundance of β-sheet hydrogen bonds relative to designs 4 and 5. The design 3 FTIR spectrum had a strong peak at 1648 cm^−1^ consistent with a random coil and a very weak peak at 1620 cm^−1^ when compared to the spectrum of the design 2 pair, suggesting an even lesser abundance of β-sheet hydrogen bonds than the other successful designs (fig. S8). Taken with the TEM images, this indicates that DMD correctly identified that design 3 would not coassemble into β-sheet fibrils. The spectrum of design 6 had a shoulder at 1620 cm^−1^ and a maximum between 1647 and 1642 cm^−1^, which, together, suggested that this peptide pair preferentially adopted random coil conformations. The absence of a strong β-sheet signal in design 6 FTIR samples suggested that the nonfibrillar oligomers observed in TEM micrographs lacked the considerable backbone hydrogen bonding associated with β-sheet structures (fig. S7). The FTIR spectrum of design 1 had a predominant maximum between 1647 and 1642 cm^−1^ and only a very weak shoulder at 1620 cm^−1^, indicating that most of the peptides in the mixture adopted random coil conformations. Notably, this suggested that the few nanofibers that are observed in transmission electron micrographs of design 1 are likely rare relative to those peptides that are unassembled or part of nonfibrillar aggregates.

**Fig. 5.**
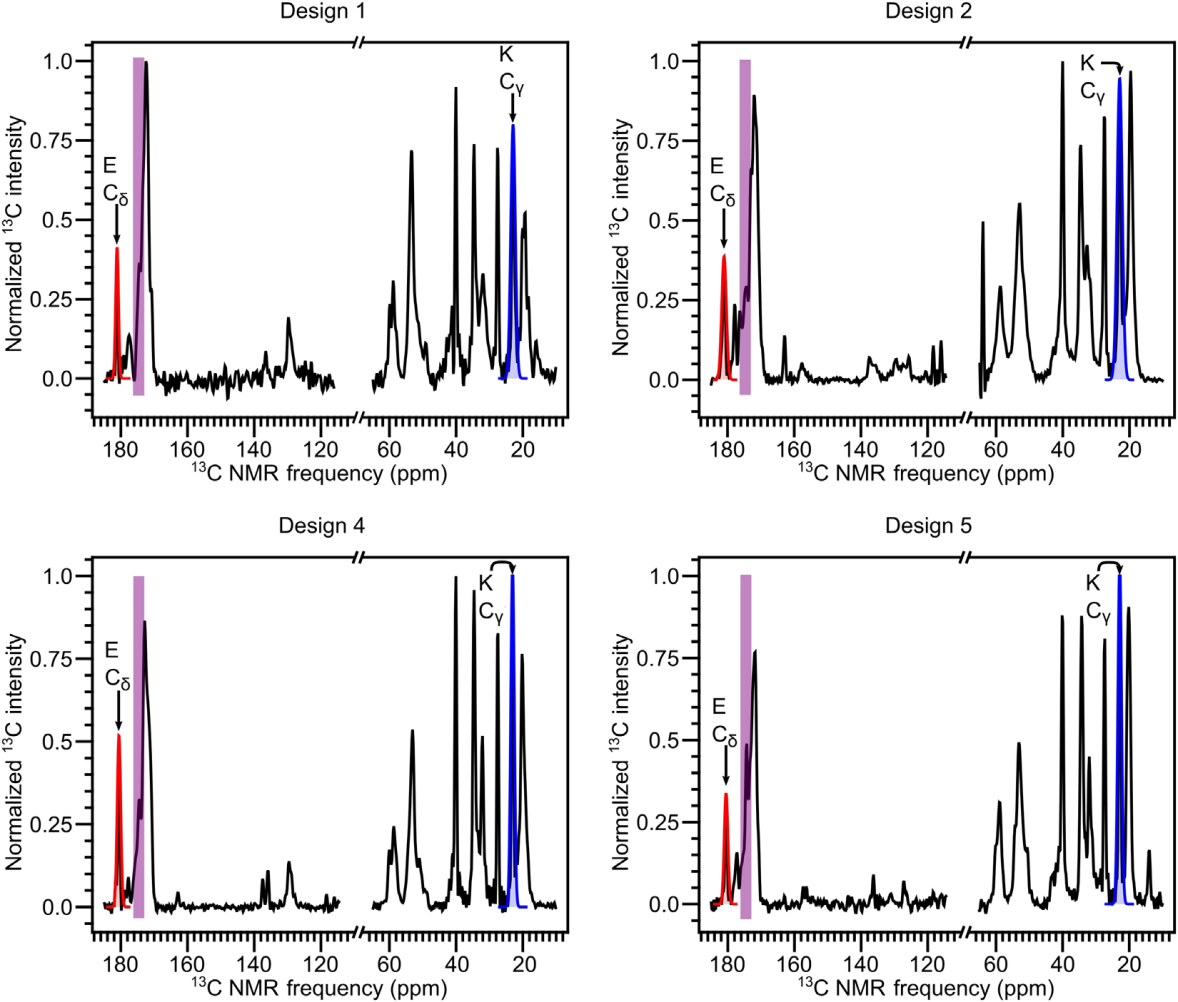
^13^C NMR spectra of centrifuged and lyophilized nanofiber samples prepared from equimolar mixtures of designs 1, 2, 4, and 5. The region highlighted in purple represents the range of carbonyl carbon chemical shift values for the two peptides in random coil conformations. The peaks highlighted in blue and red correspond to the C_γ_ of the K side chain and the C_δ_ of the E side chain.

Informed by the TEM images and FTIR measurements, designs 1, 2, 4, and 5 were further evaluated for coassembly behavior by solid-state NMR measurements on coassembled samples. Designs 3 and 6 were excluded from solid-state NMR analysis due to the lack of nanofibers in the TEM images and a mostly random coil signature in FTIR spectra. Peptide A has a distinct chemical shift peak around 23 parts per million (ppm) uniquely attributed to the γ carbon (C_γ_) of the K side chain. Similarly, peptide B has an identifiable chemical shift peak near 181 ppm uniquely assigned to δ carbon (C_δ_) of E side chain. In Fig. 5, 1D ^13^C NMR spectra of designs 1, 2, 4, and 5 all exhibit peaks at ~23 and ~181 ppm, indicating that peptides A and B are present in appreciable amounts within nanofiber samples. Thus, peptides A and B coassemble into two-component nanofibers in all four tested designs. The upfield shift in the measured CO chemical shifts as compared to the value for the same sites in a random coil conformation (Fig. 5, purple shaded region) indicates a β-strand conformation, as was also observed by FTIR and predicted by DMD/PRIME20. Together, designs 1, 2, 4, and 5 from the initial six designs successfully show selective coassembly into β sheet–rich nanofibers as originally designed and predicted by simulations.

### Computationally designed coassembling peptides show improved structural homogeneity

The ratio (relative abundance) of cationic peptide A to anionic peptide B in the four coassembled structures was determined using solid-state NMR measurements. The 1D ^13^C NMR spectra in Fig. 5 were collected in a quantitative manner allowing comparison of chemical shift peak areas. The ratio of peptide A to peptide B is reported in Table 2 for designs 1, 2, 4, and 5, as calculated from the K C_γ_ and E C_δ_ peak areas as detailed in section S10. Peak linewidths are also shown in Table 2 and are discussed in the following paragraph. The positively charged peptide A is slightly more abundant than the negatively charged peptide B in all tested pairs, consistent with our previous studies on similar coassembling β-sheet peptides. Therefore, peptides A and B are likely to arrange in a predominantly alternating (AB)*_n_* pattern, although some self-association may occur. Compared to previous designs such as the CATCH(4+/4−) design, the ratio of the two peptide components is closer to unity as shown in Table 2. This improvement in (AB)*_n_* alternation may result from the contribution of the aggregation propensity to the score function, which disfavors peptide self-association.

**Table 2.** Nanofiber composition and peak linewidth analysis for the computationally identified peptides.

	**Ratio of** **peptide A to** **peptide B**	**K C_γ_ linewidth** **in ppm** **(peptide A)**	**E C_δ_ linewidth** **in ppm** **(peptide B)**
CATCH(4+/4−)	2.22	1.098 ± 0.088	0.990 ± 0.065
Design 1	1.73	0.682 ± 0.110	0.430 ± 0.120
Design 2	1.55	0.775 ± 0.064	0.687 ± 0.145
Design 4	1.53	0.522 ± 0.065	0.526 ± 0.088
Design 5	1.78	0.553 ± 0.063	0.553 ± 0.083

Measurements of the peak linewidths in 1D ^13^C NMR spectra of designs 1, 2, 4, and 5 are compared to previous designs, indicating exceptionally well-ordered nanofibers. Linewidths (full width at half maximum) of the E C_δ_ and K C_γ_ chemical shift peaks are reported in Table 2. Broad linewidths can result from the presence of multiple distinct structures or a disordered structure. In contrast, the linewidths observed in nanofibers produced from designs 1, 2, 4, and 5 are narrow and similar to those observed in protein crystals (0.6 ppm), indicating a very highly ordered structure. Compared to linewidths in the family of CATCH peptides and King-Webb peptides (KW+, Ac-KKFEWEFEKK-Am; KW−, Ac-EEFKWKFKEE-Am) (more than 1 ppm) (*26*, *27*), the linewidths of the computationally identified pairs are almost two times smaller, suggesting that the computationally designed peptide pairs may be better behaved and produce more structurally homogeneous nanofibers.

## DISCUSSION AND CONCLUSION

Here, a computational and experimental protocol is reported to design pairs of charge-complementary peptides that can selectively coassemble into β-sheet nanofibers when mixed together but remain unassembled when isolated separately. A PepCAD algorithm was developed to discover potential selective coassembling peptides in a fast and efficient manner. The PepCAD algorithm uses a newly built score function, Γ_score_, to measure the binding free energy of the coassembling peptides A and B, as well as their intrinsic self-aggregation propensities. A lower negative value of Γ_score_ during the process of sequence evolution means that the in silico discovered peptides A and B are more likely to form fibril-like coaggregates but not fibril-like self-aggregates. As a result, six pairs of charge complimentary coassembling peptides with the lowest Γ_score_, viz. designs 1 to 6, were identified from a library of ~10^6^ candidate pairs using the PepCAD algorithm. DMD/PRIME20 simulations were then conducted to examine the co- and self-association kinetics of the six in silico peptide pairs. Designs 1, 2, 4, 5, and 6 formed amyloid-like structures after 5 μs of simulation time, whereas design 3 did not coassemble. Subsequently, the five peptide pairs were synthesized and purified, and their coassembly versus self-association was examined using TEM, FTIR, and solid-state NMR. Designs 2, 4, and 5 successfully coassembled into β-sheet nanofibers and did not self-associate; design 1 formed a combination of β-sheet nanofibers and nonfibrillar aggregates, whereas design 6 failed to form β sheet–rich structures. Designs 1, 2, 4, and 5 had solid-state NMR spectra with narrower linewidths and improved ratios of cationic to anionic peptide than the empirically designed charge-complementary coassembling peptide pairs, CATCH(+/−) and KW, confirming that the designed peptides exhibit a higher degree of structural order. This improved structural precision, coupled with the observation that none of the designed peptides aggregated when alone, highlights the accuracy of the newly developed Γ_score_ as a predictor of co- versus self-assembly propensity.

Collectively, these observations demonstrate the potential of the PepCAD algorithm for designing coassembly peptides from an experimentally intractable sequence space. In this design, our first effort at discovering 11-mer coassembling peptide pairs achieved a respectable success rate of 67%, meaning four of the six top-scoring peptides coassembled and did not self-assemble in our experiments. This is encouraging. In the future, we plan to further examine/improve the performance of the PepCAD algorithm in PepCADs, e.g., by adjusting the lengths of peptides, the combinations of (+/−) charges, and the hydrophobic/hydrophilic sequence patterns. We will also try to determine what the success rate would be if we just consider candidates that have emerged from the design stage or from the design plus kinetic simulation stages.

One lesson learned here is that designing peptide sequences to coassemble is not as straightforward as one might think. Our early design concept—to create charge-complementary peptide pairs that selectively coassemble into amyloid fibrils—was informed by the thinking that opposite and highly charged peptides should resist self-assembly due to electrostatic repulsion and coassemble through electrostatic attraction. Computational and experimental observations with coassembling peptide pairs derived from molecules known to self-assemble demonstrate that simply mixing two peptides with a high degree of opposing (i.e., attractive) charges may speed up the aggregation kinetics, but it does not guarantee exquisite molecular-level coassembly into β-sheet nanofibers (*47*). A progressive increase in the magnitudes of the opposite charges on the peptide pairs might decrease the binding free energy due to an overwhelming increase in the desolvation penalty (*48*, *49*). To capture polarization effects caused by the highly charged residues, we introduced a variable internal dielectric constant model (*50*, *51*) into the score function of PepCAD to calculate the electrostatic energy and polar solvation energy. By this way, we avoided overestimation of charge-charge interactions in this work. Although the individual peptides generally adopt a β-strand architecture when combined, like-charged neighboring strand imperfections are common and structural polymorphisms are observed (*26*, *27*). This occurs even when the CATCH(+/−) sequence pattern, PPPHPHPHPPP, where H and P refer to hydrophobic and polar amino acids, is imposed.

The PepCAD algorithm adds a much-needed layer of biophysical sophistication to these simple but appealing ideas because it accounts for the complexity in side chain–side chain interactions, which is impractical through iterative experimentally driven design processes. For example, the PepCAD algorithm has the ability to bias the fibrillar structure to be parallel/antiparallel within and between sheets. Toward this end, the algorithm preferentially designed peptides with cationic residues at the N terminus and anionic residues at the C terminus, in stark contrast to the CATCH(+/−) and KW pairs, wherein charged residues are distributed either evenly or in a core/flank arrangement. Furthermore, the PepCAD algorithm can consider a richer diversity of the naturally occurring amino acids. As a result, the algorithm preferentially designed peptides with five charged residues, used a combination of glutamic acid and aspartic acid in the anionic molecule, and placed threonine or asparagine residues at hydrophilic sites. These choices are considerable deviations from the CATCH(+/−) and KW pairs, which included four, six, or seven charged residues, only used glutamic acid, and exclusively placed charged residues or glutamine residues in hydrophilic positions.

An additional advantage of the PepCAD algorithm is that it enables us to achieve a “structure-to-sequence” design, viz. an inverse design to identify potential peptide sequences for a desired fibril-like supramolecular architectures. The performance of these types of algorithms has been analyzed by Green (*52*), who described a statistical framework for analyzing the performance of hierarchical molecular design methods. In future work aimed at improving PepCAD this statistical framework will be used to evaluate the efficiency of peptide design and predict the accuracy of its score function. Our current PepCAD is based on a fixed peptide backbone scaffold, thereby causing an inevitable bias to sequence evolution. Introducing configurational optimization to relax the peptide scaffold in PepCAD might facilitate better contacts between residues and promote the stability of fibrils assembled by designed peptides. Hopefully, a new version of PepCAD will enable the efficient design of peptides that assemble into some of the amyloid classes predicted by Sawaya *et al*. (*12*).

The procedures presented here can be thought of as a “funnel” of computational and experimental nominal yes/no tests that allow one to screen a large initial set of candidates to discover pairs of selective coassembling peptides as illustrated in Fig. 1. The funnel can also be viewed as an inverse design strategy in that the initial set of candidates is not completely random. It has been chosen to have the same length and HP sequence pattern as the CATCH(4+/6−) pairs and to form the two-layer amyloid configuration. (A difference is that each member of the pair must have five charged residues.) The funnel/inverse design strategy can, in principle, be used to screen a larger (more random) sequence space, depending on the desired outcome. In step 1, the funnel is filled with as many candidates as possible that satisfy preconceived notions such as charge complementarity and HP pattern along the chain. The PepCAD algorithm narrows this down by finding pairs whose packing energies and self-aggregation propensities are minimized for a specific structure (e.g., two stacked antiparallel β sheets). In step 2, DMD/PRIME20 simulations test whether the pairs coassemble but do not self-assemble in a reasonable time frame, 5 μs. Pairs that fail this test are rejected. In step 3, the peptides are synthesized, purified, and then subjected to biophysical characterization measurements like thioflavin T (ThT) fluorimetry, FTIR, and solid-state NMR. Peptides that fail the early tests in step 3, or are too hard to work with, are rejected. While this funnel protocol worked well, we should point out that peptides that pass step 1 do not always pass step 2, etc. For example, DMD/PRIME20 simulations suggested that design 6 could coassemble into a bilayer β sheet, albeit more slowly than the other designs, yet biophysical experiments demonstrated that the design 6 peptides aggregate but do not assemble into β-sheet nanofibers over a month at room temperature (fig. S7). Nevertheless, the protocol is highly promising.

## MATERIALS AND METHODS

Method descriptions on DMD simulation and PRIME20 force field are given in section S1. Analysis of the structure of the simulated coassembled CATCH fibril and construction of peptide scaffold of a two-layer fibril model are described in sections S2 and S3. Details regarding the use of PepCAD algorithm to de novo design coassembling peptide pairs are described in section S4. The calculations of score function, binding free energy, and intrinsic self-aggregation propensity are given in section S5. Atomistic MD simulations are performed to examine the thermodynamics stability of the in silico peptide pairs, and the amyloidogenicity of single peptide species is predicted using the FoldAmyloid Web server, as detailed in section S6. DMD/PRIME20 simulations are conducted to examine the co-/self-association kinetic of the in silico discovered peptide pairs, as detailed in section S7. Nanofiber formation from peptide coassemblies was observed on a FEI Tecnai Spirit transmission electron microscope as described in section S8. Secondary structure analysis of peptide self- and co-assembly propensity was performed using a PerkinElmer FTIR spectrophotometer as detailed in section S9. Quantitative 1D ^13^C spectra were collected for nanofiber samples on an 11.75-T Bruker Avance III spectrometer with a 3.2-mm Bruker magic angle spinning probe. NMR sample preparation and pulse sequence parameters are described in more detail in section S10. Custom code in Wolfram Mathematica was used for chemical shift peak analysis, with further discussion in section S10.

## Supplementary Materials

### This PDF file includes:

Sections S1 to S10 Table S1 Figs. S1 to S8 References

## Notes

View/request a protocol for this paper from *Bio-protocol*.
