# Supplementary material for: De novo design of peptides that coassemble into β sheet–based nanofibrils
Source: Sci Adv. 2021 Sep 3;7(36):eabf7668. doi: 10.1126/sciadv.abf7668 (PMC8442925; doi:10.1126/sciadv.abf7668)
Supplement: Supplementary file 1 — Sections S1 to S10 Table S1 Figs. S1 to S8 References [file sciadv.abf7668_sm.pdf]

Science Advances
AAAS

Supplementary Materials for

De novo design of peptides that coassemble into β sheet–based nanofibrils

Xingqing Xiao, Yiming Wang, Dillon T. Seroski, Kong M. Wong, Renjie Liu, Anant K. Paravastu, Gregory A. Hudalla, Carol K. Hall*

*Corresponding author. Email: hall@ncsu.edu

Published 3 September 2021, *Sci. Adv.* **7**, eabf7668 (2021)
DOI: 10.1126/sciadv.abf7668

This PDF file includes:

Sections S1 to S10
Table S1
Figs. S1 to S8
References

1. Discontinuous molecular dynamics (DMD) simulation and PRIME20 model

Our simulation method is discontinuous molecular dynamics (DMD), a fast alternative to traditional molecular dynamics. It is used in conjunction with PRIME20, an implicit-solvent coarse-grained protein force field developed in the Hall group, which is tailored to simulations of peptide aggregation using DMD. In the PRIME20 model, each residue contains three backbone beads NH, Cα, CO and one sidechain sphere R. The parameter matrix between any two of the twenty different sidechain-sidechain interactions include 210 different square well widths and 19 different square well depths to discriminate the polar, charge-charge and hydrophobic types of interactions. The hydrogen bonding interaction between backbone beads NH and CO is modeled as a directional square well potential. All the other non-bonded interactions are modeled as hard sphere potentials. A detailed description of the geometric and energetic parameters of the PRIME20 model is provided in our earlier work[36,39].

2. Analysis of the structure of the simulated co-assembled CATCH fibril

The specific sequences of CATCH(4+) and CATCH(6-) peptides are given in Supplementary Fig. S1a. Note that the peptides are modeled as being end-capped; thus there is no inherent preference for antiparallel arrangements in the β-sheets. Our DMD/PRIME20 simulation results showed that the CATCH peptides preferentially co-assemble into a highly-ordered fibril structure with two layers of β-sheets, as seen in Fig. 1a. The structure of this fibril can be characterized in terms of the orientations of the peptides relative to each other. Each peptide in the fibril has three levels of neighbors: (1) the nearest neighbor peptide in the same β-sheet, (2) the second nearest neighbor in the same β-sheet, and (3) the nearest peptide on the neighboring β-sheet layer. The three types of neighbors can be in four possible states: a central peptide can have a peptide neighbor that has the same charge and is either parallel or antiparallel, or the opposite charge and is either parallel or antiparallel. In Fig. 1b, we regard CATCH(6-) as the central peptide. The three groups of columns represent the three levels of neighbors described above: 1st neighbor of CATCH(6-) , 2nd neighbor of CATCH(6-), and nearest peptide of CATCH(6-) on neighboring sheet. The four columns within each group signify when a (+) is parallel to the central (-) (magenta), when a (+) is anti-parallel to the central (-) (blue), when a (-) is parallel to a central (-) (orange), and when a (-) is antiparallel to a central (-) (green). The height of the bars is the "probability" of each of the twelve possible cases. From Fig. 1b, for a CATCH(6-) peptide in the fibril, its nearest and 2nd nearest neighbor are most likely to be a CATCH(4+) that is anti-parallel and a CATCH(6-) peptide that is parallel, respectively. Also, the nearest peptide in the neighbor sheet is most likely to be a CATCH(6-) peptide and parallel. Based on the above structural analysis of the fibrillar structure formed by the mixture of CATCH(4+) and CATCH(6-) peptides, we conclude that the CATCH(+/-) peptides prefer to co-aggregate into a 2-layers with anti-parallel orientation within each β-sheet and parallel orientation between the sheets.

3. Construction of the scaffold for a 2-layer fibril model

In this work, an initial scaffold is required in the computational algorithm to discover peptide pairs that can co-assemble into a fibrillar peptide-based nanofiber in solution. We set up the desired scaffold to mimic the co-assembled fibril structure formed by CATCH peptides. DMD simulations for the co-aggregation behavior of the CATCH peptides reveals that the peptides CATCH(4+) and CATCH(6-) preferentially form a 2-layer anti-parallel fibril structure. Thus, using the Discovery Studio 3.5 software, we construct a model 2-layer fibril structure where each β-sheet layer contains four CATCH peptides (two for + and two for -) aligned in an anti-parallel orientation. The distance between the two layers is set to 12.0 Å, which is quite close to the spacing from our DMD simulation calculation and other experimental measurements. Next, the 2-layer fibril structure of the CATCH peptides is optimized in a 10-ns explicit-solvent atomistic molecular dynamics (MD) simulation using the AMBER14 package to eliminate unfavorable atomic overlaps. This optimized 2-layer fibril structure is used as the initial structure in our computational algorithm to design other co-assembled peptide pairs.

4. ***Pep*tide *c*o-*a*ssembly *d*esign (PepCAD) algorithm**

The *Pep*tide *c*o-*a*ssembly *d*esign (PepCAD) algorithm is used to *de novo* design charge-complementary peptide pairs that co-assemble into particular supramolecular architectures, e.g., a single or multiple *β*-sheet fibril (flat or twisted, parallel or antiparallel, in-register or out-of-register), a *β*-barrel oligomer, or an α-helix bundle (Supplementary Fig. S2a). A pre-determined molecular architecture, hereafter referred to as the "peptide scaffold", can be of any type, and can contain any number of assembling peptides. Supplementary Fig. S2b shows a flowsheet of the PepCAD algorithm: a Monte Carlo-based search to design co-assembling peptides A and B. The procedure is described briefly here.

(1) Random initial sequences, $S_A^{(0)}$ and $S_B^{(0)}$, are draped on the peptide backbone scaffold to generate two peptides, A and B.

(2) A score function, $\Gamma_{score}^{(0)}$, for this initial structure is introduced that takes into account the binding affinity between the peptides A and B, as well as the intrinsic aggregation propensities of the individual peptides. Scores with low (negative) values indicate that the evolved peptides have a strong preference for co-assembly and exhibit weak self-assembly behaviors. The score function will be described below.

(3) Three different kinds of trial moves, *viz*. intra-chain residue mutation, intra-chain residue exchange, and inter-chain residue exchange, are employed in each iteration, *i*, of the algorithm to mutate the peptide sequences. A random number is called at each iteration *i* to determine which type of move is employed to generate new sequences, $S_A^{(i)}$ and $S_B^{(i)}$.

(4) Energy minimization is performed to optimize the side-chain configurations of the new sequences, strengthening the binding between the new peptides A and B.

(5) The score $\Gamma_{score}^{(i)}$ for the two new peptide sequences, $S_A^{(i)}$ and $S_B^{(i)}$, is calculated and the new peptide sequences are accepted or rejected using the MC Metropolis sampling method.

(6) Steps (3), (4) and (5) are repeated 10,000 times. The peptides with the lowest scores are the best candidate sequences for peptides A and B in this design.

Three different kinds of sequence moves are used to perturb the peptide sequences, resulting in new trial peptides, A and B (Fig. 2c). The peptide configurations are kept fixed within the specified scaffold. These are: (i) *intra-chain residue mutation* in which an old residue on all trial A (or B)

peptides is replaced by a new one of the same type (hydrophobic, hydrophilic or charged) (Fig. 2c, top), (ii) *intra-chain residue exchange* in which two residues of the same type (polar "P" or hydrophobic "H") on all trial A (or B) peptides are exchanged (Fig. 2c, middle), and (iii) *inter-chain residue exchange* in which a residue of a given type on the A peptides is exchanged with a residue of the same type on the B peptides (Fig. 2c, bottom). New trial peptides A and B are generated no matter which sequence move is called in the algorithm.

The twenty standard amino acids are classified into four residue types according to their hydrophobicity, charge, hydrophilicity and structure (Supplementary Table S1). Through adjusting the number of residues in each type, we can vary the hydration properties of peptides. The CATCH(4+) and CATCH(6-) peptides that are of interest to us each contain three hydrophobic residues ($N_{\text{hydrophobic}} = 3$), three hydrophilic residues ($N_{\text{hydrophilic}} = 3$), and no other types of residue ($N_{\text{other}} = 0$). The difference between the two CATCH peptides lies in the number of charged residues: four for CATCH(4+) ($N_{\text{charge}} = 4$) and six for CATCH(6-) ($N_{\text{charge}} = 6$). In this work, our co-assembly peptide designs are restricted to $N_{\text{hydrophobic}} = 3$, $N_{\text{charge}} = 5$, $N_{\text{hydrophilic}} = 3$, and $N_{\text{other}} = 0$. During the process of sequence evolution, the number of residues in each type is fixed to maintain the evolved peptides at a desired hydration property. In this work, the sequence pattern of our designed peptides is fixed at "PPPHPHPHPPP" (Supplementary Fig. S1a), where "H" refers to hydrophobic amino acids, and "P" includes both charged and hydrophilic amino acids.

5. Score function, binding free energy and intrinsic self-aggregation propensity

Score function

The score function, an essential component of our algorithm, is built to encourage the evolved peptides A and B to co-assemble into amyloid fibrils, and to discourage them from self-assembling when dissolved separately in solution. These two factors are taken into account simultaneously in the algorithm by introducing two energy terms into the score function

$$\Gamma_{score} = \left(\Delta G_{binding} + \lambda \times P_{agg}\right)/n_{peptides} = \Delta\tilde{G}_{binding} + \lambda \times \tilde{P}_{agg} \quad (1),$$

where $\Delta G_{binding} = G^{\mathrm{AB}} - G^{\mathrm{A}} - G^{\mathrm{B}}$ is the binding free energy between all of the peptides A and B in the scaffold, P_{agg} accounts for the sum of the intrinsic self-aggregation propensity of peptide *i* (*i*=A and B), λ is a weighting factor that is used to adjust the importance of the self-aggregation propensity of the peptides during the sequence evolution process, and $n_{peptides}$ is the number of peptides (in this work, $n_{peptides} = 8$). Here, we calculate the binding free energy per peptide ($\Delta\tilde{G}_{binding}$) and intrinsic self-aggregation propensity per peptide ($\tilde{P}_{agg}$) in order to eliminate the dependence on the size of the system. A lower negative value of $\Delta G_{binding}$ indicates a stronger binding strength between peptides A and B, while a lower P_{agg} indicates a weaker aggregation propensity of the peptides in solution. Thus, a good balance between optimizing the peptides' tendency to co-aggregate and reducing their tendency to self-aggregate depends on the value chosen for λ. The settings of $\lambda = 3.0$ and 4.0 in this work are empirical, based on a quick test of values of λ to analyze the diversity of sequence variants and observe the fluctuation in the score profiles. A too-low value of λ, e.g. 0.3, restricts the types of residues in the mutation/exchange moves to certain long-sidechain amino acids that contribute too much binding energy, reducing the diversity of sequence variants. In contrast, a too-high value of λ, e.g. 30, makes it hard to ensure the convergence of sequence searches to an energy-minimum state, leading to a too-large fluctuations of the score profiles. Lower negative values of Γ_{score} means that peptides A and B are more likely to form fibril-like co-aggregates, but not fibril-like self-aggregates.

Binding free energy

The binding free energy ($\Delta G_{binding}$) accounts for the difference in the interactions between the pairs of peptides A and B when assembled in the scaffold and that when they are separated, and is defined as

$$\Delta G_{binding} = \sum(G^{AB} - G^{A} - G^{B}) \qquad (2).$$

The notation A and B are used to represent two peptides A and B, respectively. The G^{AB} indicates the free energy of the pair of peptides A and B in solution, and the G^{A} and G^{B} indicate the free energies of each individual peptide A and B, respectively. The sum is over all the pairs of the peptides A and B in the scaffold.

The free energy (G) of a molecule in the solution is calculated based on the Molecular Mechanics/Generalized Born Surface Area (MM/GBSA) method, an implicit solvent approach that combines molecular mechanics calculation and continuum solvation model and has been used widely in the atomistic MD simulations:

$$G = H - TS = \left(U_{int} + U_{vdw} + U_{ele} + G_{egb} + G_{gbsur}\right) - TS_{conf} \qquad (3),$$

where U_{int}, U_{vdw}, U_{ele}, G_{egb}, G_{gbsur}, and TS_{conf} indicate the internal energy, van der Waals energy, electrostatic energy, polar solvation energy, non-polar solvation energy and configurational entropy. The internal energy U_{int} is associated with the vibration of the bonds, bond angles, and dihedral angles. The van der Waals energy U_{vdw} is calculated using the typical 12-6 Lennard-Jones equation. The electrostatic energy U_{ele} follows Coulomb's law. The polar solvation energy G_{egb} is calculated based on the Generalized Born method with the Onufriev-Bashford-Case model. The nonpolar solvation energy G_{gbsur} is proportional to the solvent-accessible surface area of solute molecule. The configurational entropy TS_{conf} is estimated by using the quasi-harmonic approximation. The expressions for U_{int}, U_{vdw}, U_{ele}, G_{egb}, and G_{gbsur} can be found in our previous work[31].

The configurational entropy, TS_{conf}, is

$$TS_{conf} = \frac{1}{2}nRT + \frac{1}{2}RT\ln[(2\pi)^{n} \cdot \det(\boldsymbol{\sigma})] \qquad (4),$$

where n is the number of torsional angles of standard amino acid side-chain; R is the gas constant; T is the absolute temperature; det stands for the determinant of a matrix; $\boldsymbol{\sigma}$ is the variance-covariance matrix of torsional fluctuations, whose dimension is related to the number of torsion angles (n) for a specific amino acid. For example, since the amino acid methionine has three torsion angles ($n = 3$) on its sidechain, the dimension of the matrix $\boldsymbol{\sigma}$ is $[n, n] = [3, 3]$. The element of the variance-covariance matrix, σ_{ij}, is given by

$$\sigma_{ij} = \langle(\chi_i - \langle\chi_i\rangle) \cdot (\chi_j - \langle\chi_j\rangle)\rangle \qquad (5),$$

where χ_i denotes the torsion angle of the i_{th} side-chain (i.e. $i = 1, \dots, n$), and $\langle \dots \rangle$ is an ensemble average over all possible rotamers of this amino acid when repacked during sequence evolution. The covariance σ_{ij} reflects the correlation between the side-chain torsions i and j.

The free energy G of the individual peptides A and B in equation (3) is the sum of the terms. The binding free energy $\Delta G_{binding}$ is defined to be the difference between the free energy of the peptides A and B prior to binding. A lower negative value of $\Delta G_{binding}$ indicates a stronger binding strength between peptides A and B. All of the force field parameters in the calculation of $\Delta G_{binding}$ come from the AMBER 14SB force field.

Intrinsic self-aggregation propensity

The intrinsic self-aggregation propensity P_{agg} of a polypeptide containing N sites along the chain can be estimated using the Zyggregator method proposed by the Dobson and Vendruscolo groups[43-45]:

$$P_{agg} = \delta_{hydr} I_{hydr} + \delta_\alpha I_\alpha + \delta_\beta I_\beta + \delta_{ch} I_{ch} + \delta_{pat} I_{pat} \qquad (6),$$

where I_{hydr} indicates the hydrophobicity of the sequence, I_α is the α-helical propensity, I_β is the β-sheet propensity, I_{ch} is the absolute value of the net charge of the sequence, and I_{pat} is the sequence pattern. The coefficients δ_i, which weight each of the individual factors, were obtained by fitting to a database of peptides/proteins whose aggregation rates are measured experimentally, as given below: $\delta_{hydr} = -1.0$, $\delta_\alpha = -5.7$, $\delta_\beta = 5.0$, $\delta_{ch} = -0.16$, $\delta_{pat} = 0.39$. It is worth noting that we ignore the sequence pattern term I_{pat} throughout the entire evolution process because the sequence pattern is fixed. A low negative value for P_{agg} indicates a weak aggregation propensity for the peptides in solution.

(g) Hydrophobicity of peptide sequence I_{hydr}

Assuming the polypeptide chain has $N_{\text{hydrophobic}}$ hydrophobic residues, $N_{\text{charge+}}$ positively-charged residues, $N_{\text{charge-}}$ negatively-charged residues, $N_{\text{hydrophilic}}$ hydrophilic residues, and N_{other} other residues (here, $N_{\text{hydrophobic}} + N_{\text{charge+}} + N_{\text{charge-}} + N_{\text{hydrophilic}} + N_{\text{other}} = N$), the hydrophobicity I_{hydr} of the peptide is defined as

$$I_{hydr} = \left| \sum_{i=1}^{N_{\text{hydrophobic}}} p^i - \tilde{p}_{\text{hydrophobic}} \cdot N_{\text{hydrophobic}} \right| + \left| \sum_{i=1}^{N_{\text{charge+}}} p^i - \tilde{p}_{\text{charge+}} \cdot N_{\text{charge+}} \right|$$

$$+\left|\sum_{i=1}^{N_{\text{charge-}}} p^i - \tilde{p}_{\text{charge-}} \cdot N_{\text{charge-}}\right| + \left|\sum_{i=1}^{N_{\text{hydrophilic}}} p^i - \tilde{p}_{\text{hydrophilic}} \cdot N_{\text{hydrophilic}}\right| + \left|\sum_{i=1}^{N_{\text{other}}} p^i - \tilde{p}_{\text{other}} \cdot N_{\text{other}}\right| \quad (7),$$

where p is the hydrophobicity scale of amino acid; $\tilde{p}$ is the average hydrophobicity of each residue type in natural resources: $\tilde{p}_{\text{hydrophobic}} = -1.82$, $\tilde{p}_{\text{charge+}} = 1.36$, $\tilde{p}_{\text{charge-}} = 0.95$, $\tilde{p}_{\text{hydrophilic}} = 0.10$, and $\tilde{p}_{\text{other}} = -0.61$; $|\ldots|$ is an absolute value. All the parameters regarding p and $\tilde{p}$ can be seen in references[53,54].

(f) α-helical propensity I_α, β-sheet propensity I_β, net charge I_{ch}

$$I_\alpha = \sum_{i=1}^{N} p_\alpha^i \quad (8),$$

$$I_\beta = \sum_{i=1}^{N} p_\beta^i \quad (9),$$

$$I_{ch} = \left|\sum_{i=1}^{N} p_{ch}^i\right| \quad (10),$$

where p_α and p_β are the amino acid scales for α-helix and β-sheet formation, respectively; p_{ch} is the charge of amino acid; $|\ldots|$ is an absolute value.

(i) Sequence pattern I_{pat}

$$I_{pat} = \sum_{i=1}^{N} p_{pat}^i \quad (11),$$

where p_{pat} accounts for the presence of alternating hydrophobic-hydrophilic sequence pattern. The p_{pat}^i is 1 if residue i is included in this specific sequence pattern, and 0 otherwise.

The intrinsic aggregation propensity P_{agg} of the polypeptide in equation (6) is the sum of equations (7-11). It is worth noting that the sequence pattern term I_{pat} is ignorable throughout the entire evolution process, because the sequence pattern of all evolved peptides is unchanged. A lower P_{agg} indicates a weaker aggregation propensity of the peptides in solution.

6. Atomistic molecular dynamics (MD) simulation and the FoldAmyloid web-sever

Explicit-solvent atomistic MD simulations are carried out at 298 K in the NVT ensemble using the AMBER16 package with the 14SB force field to examine the thermodynamic stability of the six 2-layer amyloid fibrils consisting of the designed peptide pairs. The starting structures of the six 2-layer amyloid fibrils are obtained from the output of the PepCAD algorithm. Each fibril structure is solvated in a periodic truncated octahedral box containing an 8 Ångstrom buffer of TIP3P water (~6000 water molecules) surrounding the fibril in each direction. No counterions are required in each system because of their neutrality.

The 12 peptides in the six designs were evaluated using the FoldAmyloid web-server (http://bioinfo.protres.ru/fold-amyloid/) to see how likely they are to self-aggregate. The FoldAmyloid method, a bioinformatics method, uses statistical data on natural amyloid proteins to obtain inherent aggregation scales for standard amino acids. It then classifies a region along a peptide chain to be non-amyloidogenic if it contains at least 7 consecutive residues that have average self-aggregation scale lower than an empirical threshold value of 21.4; otherwise the region is amyloidogenic.

7. DMD/PRIME20 simulations of co-/self-association kinetics of *in-silico* discovered peptide pairs

We performed large-scale DMD/PRIME20 simulations to evaluate the spontaneous aggregation and co-assembled structures of the six best peptide pairs designed in our PepCAD algorithm. In addition, we also performed simulations of the co-assembly of the CATCH peptide pair designed by Seroski et al.[24] All the simulations start with random-coil peptides and are carried out for 5 μs in the canonical (NVT) ensemble. The Andersen thermostat is implemented to maintain the simulation system at a constant temperature. For the peptide co-assembly cases, 100 A and 100 B peptides are initially randomly placed in a cubic box with a length of 321.0 Å, corresponding to a peptide concentration of 10 mM. The reduced temperature is defined to be $T^{*}=k_{B}T/\varepsilon_{HB}$, where ε_{HB}=12.47 kJ/mol is the hydrogen bonding energy. We set the reduced temperature T^{*} of the simulations to be 0.195, which corresponds to 330 K in real temperature units. The β-sheet content is defined as the percent of residues in the whole system that adopts β-sheet structure, which is calculated using the VMD secondary structure software. For the peptide self-assembly cases, 40 A or 40 B are simulated at the same concentration and temperature as in the co-assembly cases. Each of the peptide systems starts from a random-coil state and is simulated three times.

8. Transmission electron microscopy

Nanofibers were prepared by mixing and incubating positively charged peptides with negatively charged peptide at 1 mM final concentration overnight in 1x PBS, unless otherwise stated. Samples were diluted to 250 μM with ultrapure water followed filtration through a 0.22 μM syringe filter before loading onto grids. Formvar-carbon coated 400 mesh copper grids (FCF400-CU-UB, EMS) were floated for 30 seconds on top of 5 μl peptide nanofibers and then dried by tilting the grid on a Kimwipe. Samples were then negatively stained with 2% uranyl acetate in water for 5 mins and analyzed using a Hitachi H-700 for endpoint studies on a FEI Tecnai Spirit (FEI, The Netherlands) housed in the University of Florida Interdisciplinary Center for Biotechnology Research (ICBR).

9. Fourier-transform infrared spectroscopy (FTIR)

The FTIR spectra were recorded using a universal ATR sampling accessory on a Frontier FTIR spectrophotometer (PerkinElmer). Prior to scanning, the FTIR spectrophotometer was blanked with ultrapure water. Samples were prepared at 15 mM and 1x PBS with 4 μl spotted onto the ATR accessory. Each sample was scanned 50 times with the average of the spectra reported.

10. Solid-state NMR analysis of co-assembled nanofiber samples

For each tested design, nanofiber samples were prepared from equimolar mixtures of peptide A and peptide B at a 10 mM peptide concentration in 1x phosphate-buffered saline (PBS). Samples for Designs 2, 4, and 5 were allowed to incubate for 1 day before centrifugation at 12,100 × g for 5 minutes. Recovered nanofibers are lyophilized prior to packing into 3.2 mm NMR rotors. Finally, samples were minimally rehydrated (1 mg of water per mg of peptide). Due to the lower initial nanofiber yield, the Design 1 mixture was allowed to assemble over 4 days, and nanofibers were recovered by ultracentrifugation directly into the NMR rotor to increase sample yield. Ultracentrifugation was done at 280 000 × g and 4 °C for 30 min on a Beckman Optima XPN-100 fitted with a SW-41 Ti swinging-bucket rotor and custom-made polycarbonate funnel insert.

The composite-pule multiCP pulse sequence from Duan et al. was implemented to perform quantitative 1H-13C Cross-Polarization Magic Angle Spinning (CPMAS) measurements[55]. Quantitative CPMAS measurments were run on an 11.75 T Bruker Avance III spectrometer with 100 kHz decoupling and 14 100-μs CP periods to ensure uniform cross polarization. The spinning speed was set to 22 kHz to prevent spectral overlap from spinning sidebands. Reported chemical shifts are relative to tetramethyl silane by calibration with adamantine before each experiment.

Analysis of the chemical shift peaks was done with custom code in Wolfram Mathematica. Peak positions, linewidths, and areas were determined from Lorentzian peak fitting. Chemical shift peak assignments were done by comparison to expected chemical shift peak positions from the BioMagResBank. The ratio of peptide A to peptide B was determined as the ratio of the K γ-carbon (Cγ) peak area to the E δ-carbon (Cδ) peak area adjusted for the number of K and E residues in each sequence.

Supplementary Table S1. The classification of 20 natural amino acids into four residue types: hydrophobic, charged, hydrophilic and other. The category labeled by "H" includes the hydrophobic amino acids, while the category labeled by "P" includes the charged and hydrophilic amino acids.

Hydrophobic Residues	Ala(A), Leu(L), Val(V) Ile(I), Met(M) Phe(F), Tyr(Y), Trp(W)	3	**H**
Charged Residues	Arg(R), Lys(K) Glu(E), Asp(D)	5	**P**
Hydrophilic Residues	Asn(N), Gln(Q) Ser(S), Thr(T), His(H)	3	
Other Residues	Cys(C), Pro(P), Gly(G)	0	

(a) Site	1	2	3	4	5	6	7	8	9	10	11
CATCH(4+) **(peptide A)**	**Q**	**Q**	**K**	**F**	**K**	**F**	**K**	**F**	**K**	**Q**	**Q**
CATCH(6-) **(peptide B)**	**E**	**Q**	**E**	**F**	**E**	**F**	**E**	**F**	**E**	**Q**	**E**
**Sequence pattern**	**P**	**P**	**P**	**H**	**P**	**H**	**P**	**H**	**P**	**P**	**P**

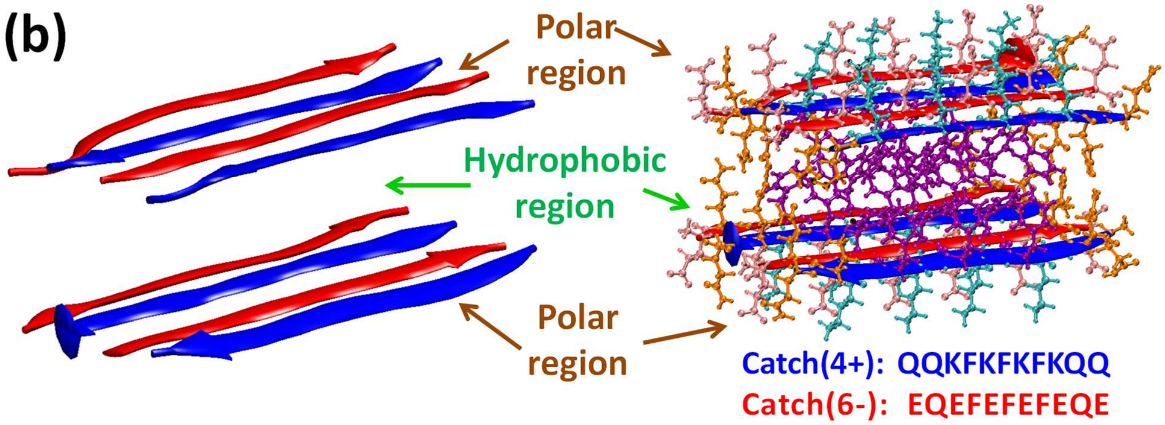

Supplementary Fig. S1. (a) The two experimentally-discovered 11-mer co-assembly peptides, CATCH(4+) with four positive charges and CATCH(6-) with six negative charges. Both peptides follow the sequence pattern: PPPHPHPHPPP. (b) A 2-layer fibril model consisting of four CATCH(4+) peptides and four CATCH(6-) peptides is constructed using the Discovery Studio 3.5 software and optimized using the AMBER14 package. Eight CATCH peptides (four for + and four for -) are aligned in an anti-parallel orientation within the sheet. The hydrophobic residues on the CATCH peptides aggregate to form a hydrophobic core sandwiched between the two β-sheet layers. Most of the hydrophilic and charged residues are exposed to the "water" to stabilize the amyloid fibril. This 2-layer structure is used as an initial conformation in the PepCAD algorithm.

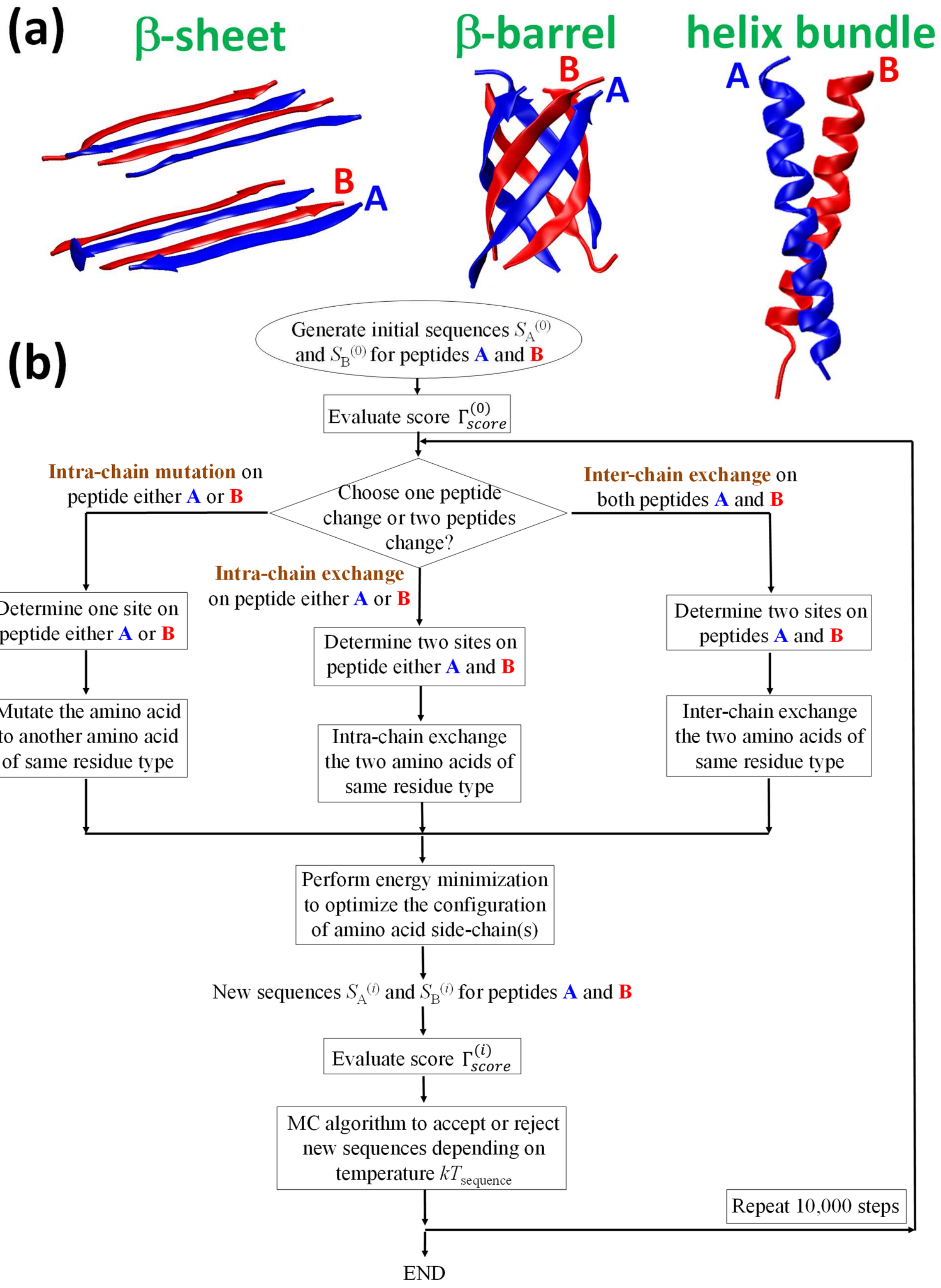

Supplementary Fig. S2. (a) Types of peptide scaffolds considered, including β-sheet fibril, β-barrel oligomer, and α-helix bundle. (b) Flow sheet of the computational algorithm that is used to design fibril-forming co-assembly peptides.

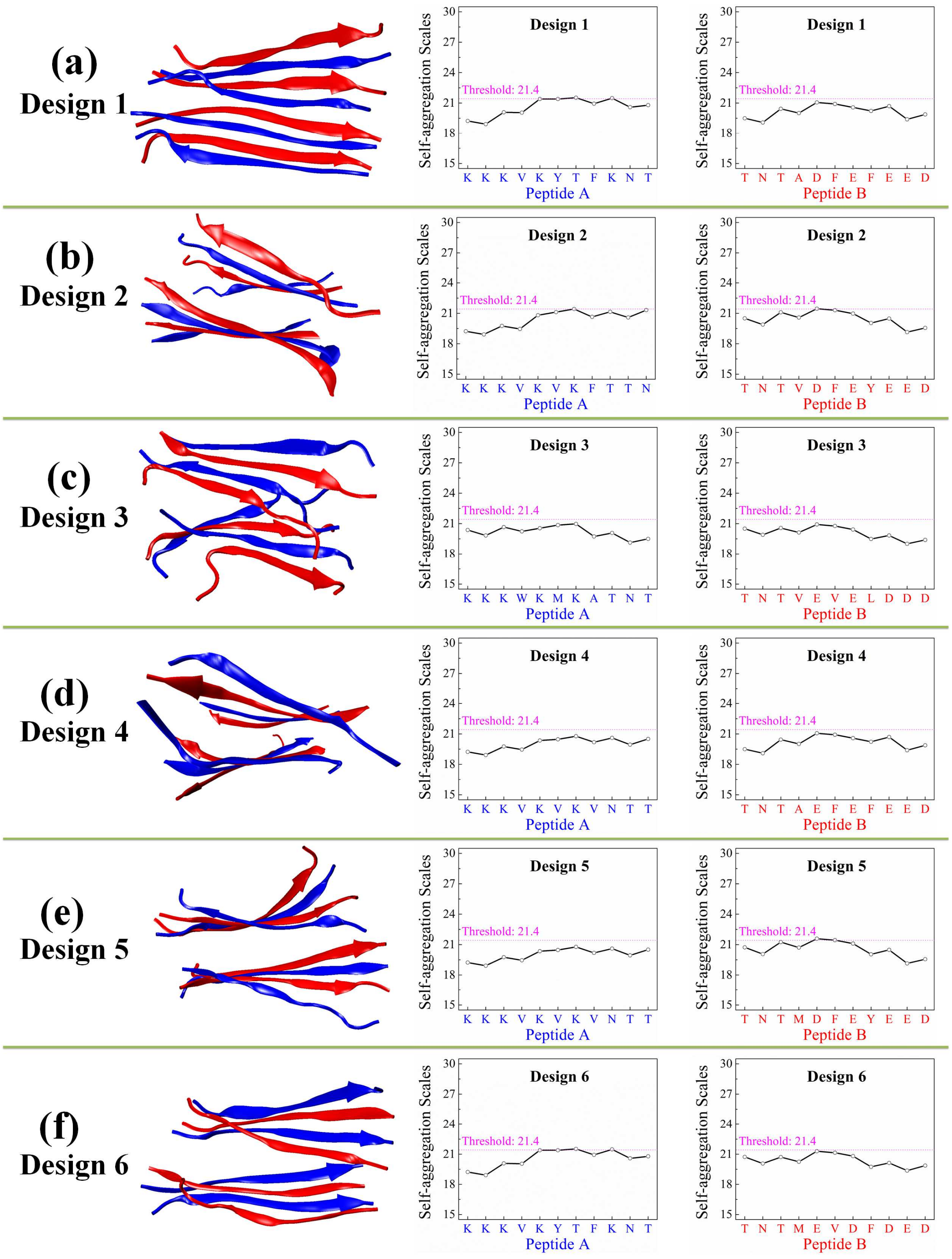

Supplementary Fig. S3. Simulated structures of the 2-layer amyloid fibrils in Designs 1-6 after 100-ns atomistic MD simulations in the AMBER16 package. The self-aggregation scales for each individual residue along with the sequences of co-assembling peptides A and B in the six pairs are evaluated using the FoldAmyloid tool.

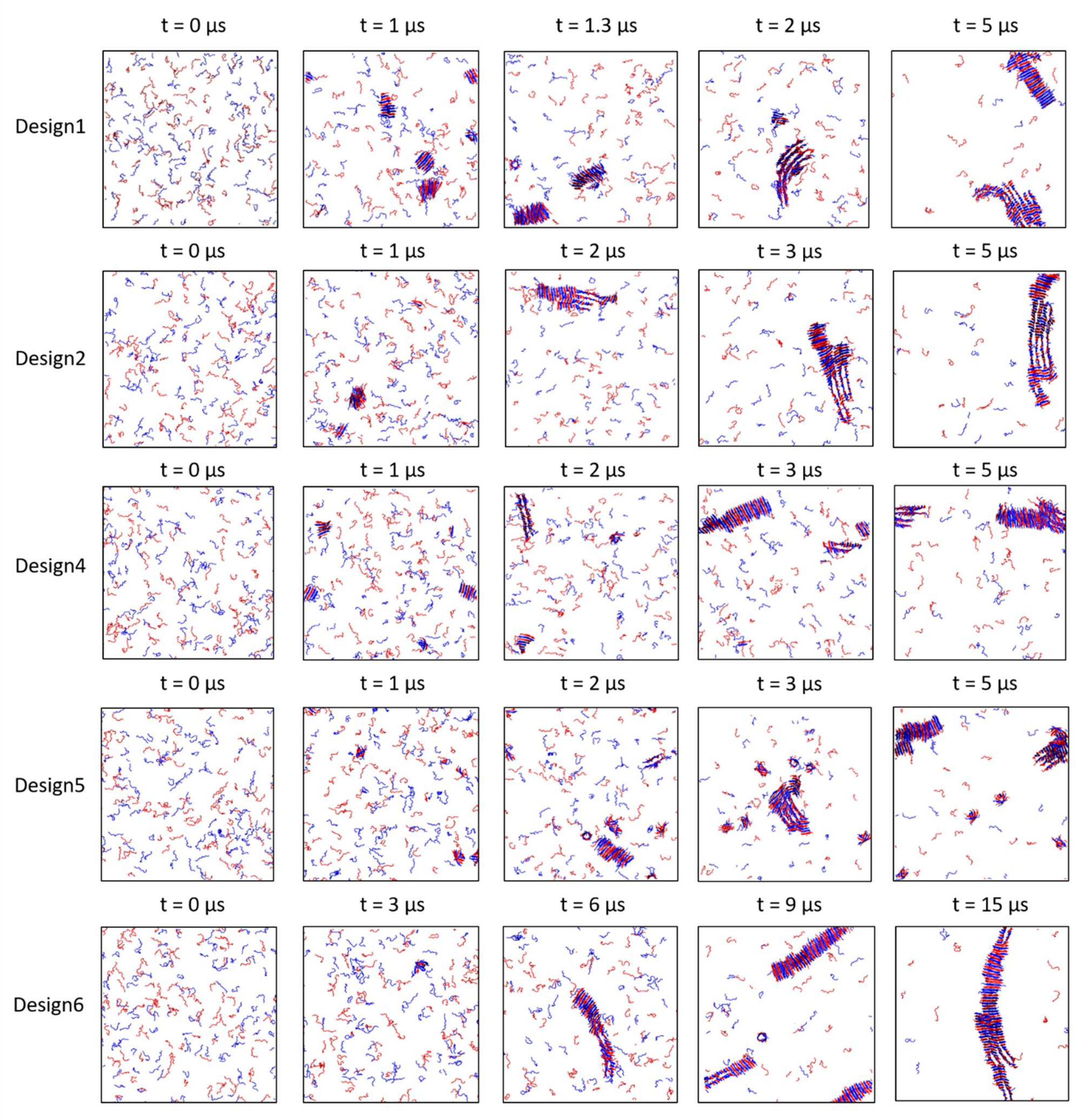

Supplementary Fig. S4. Simulation snapshots of co-aggregation trajectories of Designs 1, 2, 4, 5 and 6 peptide pairs at different time points.

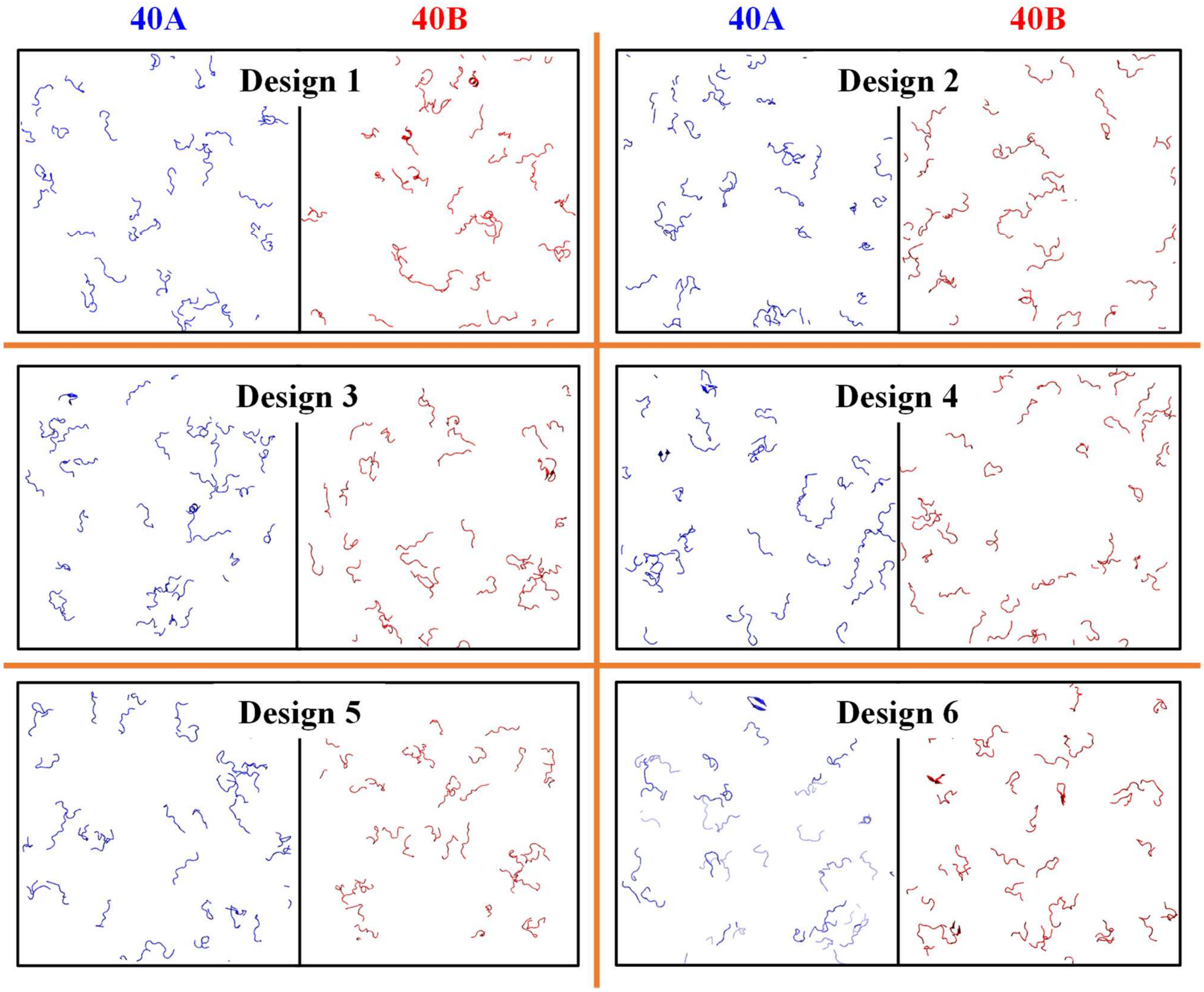

Supplementary Fig. S5. DMD/PRIME20 simulations of peptide self-aggregation for Designs (1-6) containing 40A only and 40B only.

(a)		Sequences											Scores
Initial pair 1	Peptide A	D	R	K	L	E	F	R	A	T	Q	S	7.79
	Peptide B	E	Q	H	F	D	Y	N	V	K	R	E	
Initial pair 2	Peptide A	E	T	S	Y	D	F	K	A	R	E	Q	8.85
	Peptide B	R	S	E	I	Q	W	N	L	D	E	E	
Initial pair 3	Peptide A	H	R	E	W	R	L	R	I	T	R	Q	11.51
	Peptide B	E	S	N	F	E	M	R	W	K	Q	K	

Initial pair 1 Initial pair 2 Initial pair 3

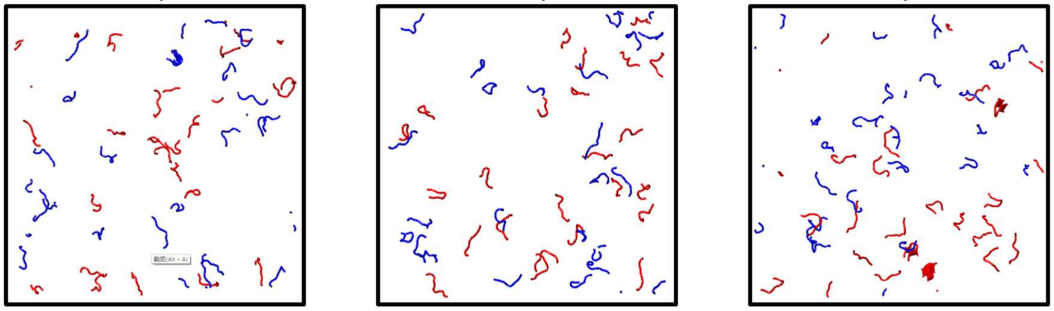

(b)		Sequences											Scores
Mid pair 4	Peptide A	K	K	K	I	R	V	R	M	S	S	T	-14.72
	Peptide B	H	T	D	F	D	W	D	A	N	E	E	
Mid pair 5	Peptide A	N	R	T	M	K	V	K	F	R	K	T	-12.43
	Peptide B	T	E	E	V	E	F	N	Y	E	D	S	
Mid pair 6	Peptide A	Q	T	K	Y	K	F	K	L	K	K	T	-12.21
	Peptide B	H	E	E	F	E	V	D	M	N	D	T	

Mid pair 4 Mid pair 5 Mid pair 6

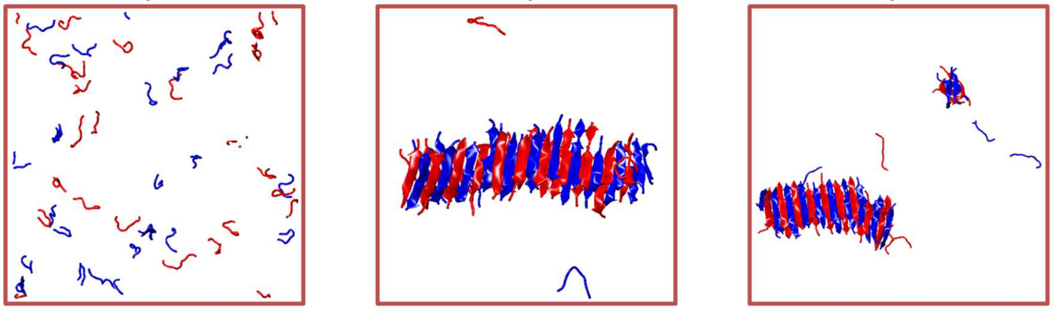

Supplementary Fig. S6. (a) Three initial random peptide pairs and (b) three *in-silico* peptide pairs in the middle of evolution with medium scores are chosen to examine their co-assembly behaviors in the DMD/PRIME20 simulations.

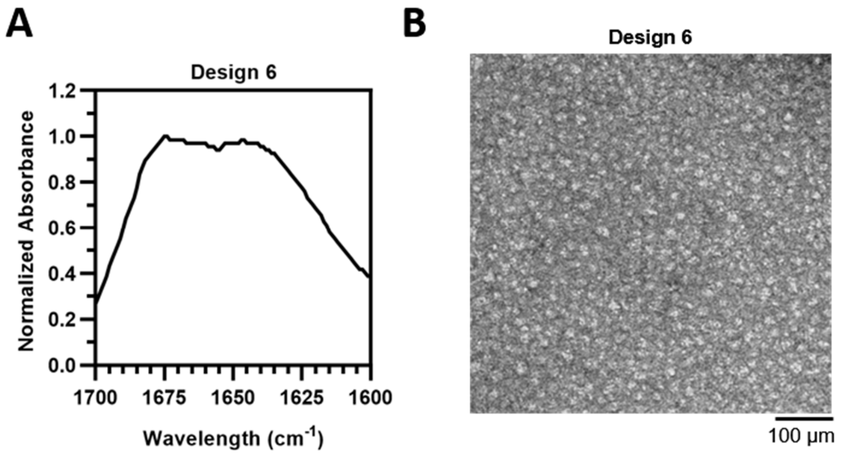

Supplementary Fig. S7. (A) FTIR spectra of Design 6 at 15 mM after 1 month of incubation in 1x PBS. (B) Transmission electron micrograph of Design 6 at 1 mM diluted with water to 0.25 mM after 1 month of incubation in 1x PBS.

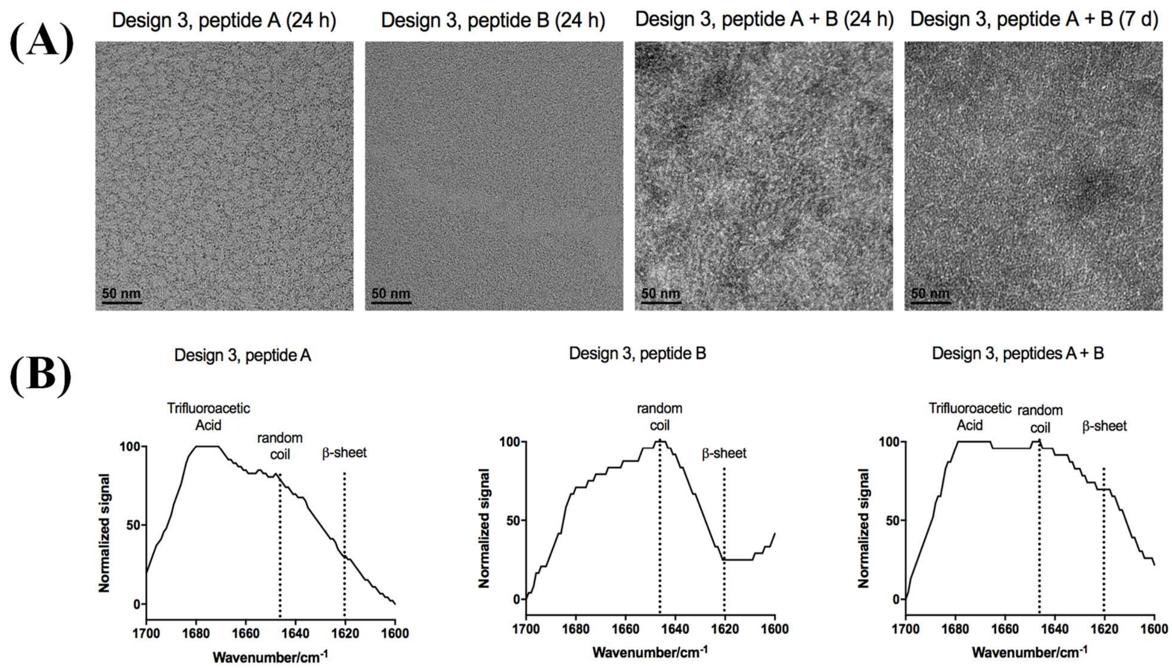

Supplementary Fig. S8. (A) TEM micrographs of Design 3, peptide A at t = 24 h, Design 3 peptide B at t = 24 h and the mixture of Design 3 peptides A + B at t = 24 h (left) and 7 days (right). (B) FTIR spectra of Design 3, peptide A at t = 24 h, Design 3 peptide B at t = 24 h, and the mixture of Design 3 peptides A + B at t = 24 h.

REFERENCES AND NOTES

1. S. Kim, J. H. Kim, J. S. Lee, C. B. Park, Beta-sheet-forming, self-assembled peptide nanomaterials towards optical, energy, and healthcare applications. *Small* **11**, 3623–3640 (2015).

2. S. Scanlon, A. Aggeli, Self-assembling peptide nanotubes. *Nano Today* **3**, 22–30 (2008).

3. J. D. Hartgerink, J. R. Granja, R. A. Milligan, M. R. Ghadiri, Self-assembling peptide nanotubes. *J. Am. Chem. Soc.* **118**, 43–50 (1996).

4. S. Zhang, Fabrication of novel biomaterials through molecular self-assembly. *Nat. Biotechnol.* **21**, 1171–1178 (2003).

5. K. Matsuura, K. Murasato, N. Kimizuka, Artificial peptide-nanospheres self-assembled from three-way junctions of β-sheet-forming peptides. *J. Am. Chem. Soc.* **127**, 10148–10149 (2005).

6. S. Vauthey, S. Santoso, H. Gong, N. Watson, S. Zhang, Molecular self-assembly of surfactant-like peptides to form nanotubes and nanovesicles. *Proc. Natl. Acad. Sci. U.S.A.* **99**, 5355–5360 (2002).

7. J. D. Hartgerink, E. Beniash, S. I. Stupp, Self-assembly and mineralization of peptide-amphiphile nanofibers. *Science* **294**, 1684–1688 (2001).

8. P. Sormanni, F. A. Aprile, M. Vendruscolo, The CamSol method of rational design of protein mutants with enhanced solubility. *J. Mol. Biol.* **427**, 478–490 (2015).

9. P. Sormanni, L. Amery, S. Ekizoglou, M. Vendruscolo, B. Popovic, Rapid and accurate *in silico* solubility screening of a monoclonal antibody library. *Sci. Rep.* **7**, 8200 (2017).

10. M. Oliveberg, Waltz, an exciting new move in amyloid prediction. *Nat. Methods* **7**, 187–188 (2010).

11. I. Walsh, F. Seno, S. C. T. Tosatto, A. Trovato, PASTA 2.0: An improved server for protein aggregation prediction. *Nucleic Acids Res.* **42**, W301–W307 (2014).

12. M. R. Sawaya, S. Sambashivan, R. Nelson, M. L. Ivanova, S. A. Sievers, M. L. Apostol, M. J. Thompson, M. Balbirnie, J. J. Wiltzius, H. T. McFarlane, A. Ø. Madsen, C. Riekel, D. Eisenberg, Atomic structures of amyloid cross-β spines reveal varied steric zippers. *Nature* **447**, 453–457 (2007).

13. S. Zhang, T. Holmes, C. Lockshin, A. Rich, Spontaneous assembly of a self-complementary oligopeptide to form a stable macroscopic membrane. *Proc. Natl. Acad. Sci. U.S.A.* **90**, 3334–3338 (1993).

14. H. Yokoi, T. Kinoshita, S. Zhang, Dynamic reassembly of peptide RADA16 nanofiber scaffold. *Proc. Natl. Acad. Sci. U.S.A.* **102**, 8414–8419 (2005).

15. S. R. Leonard, A. R. Cormier, X. Pang, M. I. Zimmerman, H. X. Zhou, A. K. Paravastu, Solid-state NMR evidence for β-hairpin structure within MAX8 designer peptide nanofibers. *Biophys. J.* **105**, 222–230 (2013).

16. C. J. Wilson, A. S. Bommarius, J. A. Champion, Y. O. Chernoff, D. G. Lynn, A. K. Paravastu, C. Liang, M. C. Hsieh, J. M. Heemstra, Biomolecular assemblies: Moving from observation to predictive design. *Chem. Rev.* **118**, 11519–11574 (2018).

17. C. G. Pappas, R. Shafi, I. R. Sasselli, H. Siccardi, T. Wang, V. Narang, R. Abzalimov, N. Wijerathne, R. V. Ulijn, Dynamic peptide libraries for the discovery of supramolecular nanomaterials. *Nat. Nanotechnol.* **11**, 960–967 (2016).

18. P. W. Frederix, G. G. Scott, Y. M. Abul-Haija, D. Kalafatovic, C. G. Pappas, N. Javid, N. T. Hunt, R. V. Ulijn, T. Tuttle, Exploring the sequence space for (tri-) peptide self-assembly to design and discover new hydrogels. *Nat. Chem.* **7**, 30–37 (2015).

19. C. A. Hauser, S. Maurer-Stroh, I. C. Martins, Amyloid-based nanosensors and nanodevices. *Chem. Soc. Rev.* **43**, 5326–5345 (2014).

20. R. N. Shah, N. A. Shah, M. M. Del Rosario Lim, C. Hsieh, G. Nuber, S. L. Stupp, Supramolecular design of self-assembling nanofibers for cartilage regeneration. *Proc. Natl. Acad. Sci. U.S.A.* **107**, 3293–3298 (2010).

21. Y. Liang, P. Guo, S. V. Pingali, S. Pabit, P. Thiyagarajan, K. M. Berland, D. G. Lynn, Light harvesting antenna on an amyloid scaffold. *Chem. Commun.* **48**, 6522–6524 (2008).

22. G. A. Hudalla, T. Sun, J. Z. Gasiorowski, H. Han, Y. F. Tian, A. S. Chong, J. H. Collier, Gradated assembly of multiple proteins into supramolecular nanomaterials. *Nat. Mater.* **13**, 829–836 (2014).

23. P. J. S. King, M. G. Lizio, A. Booth, R. F. Collins, J. E. Gough, A. F. Miller, S. J. Webb, A modular self-assembly approach to functionalised β-sheet peptide hydrogel biomaterials. *Soft Matter* **12**, 1915–1923 (2016).

24. D. T. Seroski, A. Restuccia, A. D. Sorrentino, K. R. Knox, S. J. Hagen, G. A. Hudalla, Co-assembly tags based on charge complementarity (CATCH) for installing functional protein ligands into supramolecular biomaterials. *Cell. Mol. Bioeng.* **9**, 335–350 (2016).

25. J. H. Collier, P. B. Messersmith, Enzymatic modification of self-assembled peptide structures with tissue transglutaminase. *Bioconjug. Chem.* **14**, 748–755 (2003).

26. Q. Shao, K. M. Wong, D. T. Seroski, Y. Wang, R. Liu, A. K. Paravastu, G. A. Hudalla, C. K. Hall, Anatomy of a selectively coassembled β-sheet peptide nanofiber. *Proc. Natl. Acad. Sci. U.S.A.* **9**, 4710–4717 (2020).

27. K. M. Wong, Y. Wang, D. T. Seroski, G. E. Larkin, A. K. Mehta, G. A. Hudalla, C. K. Hall, A. K. Paravastu, Molecular complementarity and structural heterogeneity within co-assembled peptide β-sheet nanofibers. *Nanoscale* **12**, 4506–4518 (2020).

28. X. Xiao, C. K. Hall, P. F. Agris, The design of a peptide sequence to inhibit HIV replication: A search algorithm combining Monte Carlo and self-consistent mean field techniques. *J. Biomol. Struct. Dyn.* **32**, 1523–1536 (2014).

29. X. Xiao, P. F. Agris, C. K. Hall, Designing peptide sequences in flexible chain conformations to bind RNA: A search algorithm combining Monte Carlo, self-consistent mean field and concerted rotation techniques. *J. Chem. Theory Comput.* **11**, 740–752 (2015).

30. X. Xiao, P. F. Agris, C. K. Hall, Introducing folding stability into the score function for computational design of RNA-binding peptides boosts the probability of success. *Proteins* **84**, 700–711 (2016).

31. X. Xiao, M. E. Hung, J. N. Leonard, C. K. Hall, Adding energy minimization strategy to peptide-design algorithm enables better search for RNA-binding peptides: Redesigned λ N peptide binds boxB RNA. *J. Comput. Chem.* **37**, 2423–2435 (2016).

32. X. Xiao, Y. Wang, J. N. Leonard, C. K. Hall, Extended concerted rotation technique enhances the sampling efficiency of the computational peptide-design algorithm. *J. Chem. Theory Comput.* **13**, 5709–5720 (2017).

33. J. L. Spears, X. Xiao, C. K. Hall, P. F. Agris, Amino acid signature enables proteins to recognize modified tRNA. *Biochemistry* **53**, 1125–1133 (2014).

34. X. Xiao, Z. Kuang, J. M. Slocik, S. Tadepalli, M. Brothers, S. Kim, P. A. Mirau, C. Butkus, B. L. Farmer, S. Singamaneni, C. K. Hall, R. R. Naik, Advancing peptide-based biorecognition elements for biosensors using *in-silico* evolution. *ACS Sens.* **3**, 1024–1031 (2018).

35. X. Xiao, Z. Kuang, B. J. Burke, Y. Chushak, B. L. Farmer, P. A. Mirau, R. R. Naik, C. K. Hall, In silico discovery and validation of neuropeptide-Y-binding peptides for sensors. *J. Phys. Chem. B* **124**, 61–68 (2020).

36. M. Cheon, I. Chang, C. K. Hall, Extending the PRIME model for protein aggregation to all 20 amino acids. *Proteins* **78**, 2950–2960 (2010).

37. M. Cheon, I. Chang, C. K. Hall, Spontaneous formation of twisted $A\beta_{16\text{-}22}$ fibrils in large-scale molecular-dynamics simulations. *Biophys. J.* **101**, 2493–2501 (2011).

38. D. C. Latshaw, M. Cheon, C. K. Hall, Effects of macromolecular crowding on amyloid beta (16-22) aggregation using coarse-grained simulations. *J. Phys. Chem. B* **118**, 13513–13526 (2014).

39. Y. Wang, Q. Shao, C. K. Hall, N-terminal prion protein peptides (PrP(120–144)) form parallel in-register β-sheets via multiple nucleation-dependent pathways. *J. Biol. Chem.* **292**, 20655 (2017).

40. Y. Wang, S. J. Bunce, S. E. Radford, A. J. Wilson, S. Auer, C. K. Hall, Thermodynamic phase diagram of amyloid-β (16-22) peptide. *Proc. Natl. Acad. Sci. U.S.A.* **116**, 2091–2096 (2019).

41. H. Gohlke, C. Kiel, D. A. Case, Insights into protein-protein binding by binding free energy calculation and free energy decomposition for the Ras-Raf and Ras-RalGDS complexes. *J. Mol. Biol.* **330**, 891–913 (2003).

42. K. F. DuBay, A. P. Pawar, F. Chiti, J. Zurdo, C. M. Dobson, M. Vendruscolo, Prediction of the absolute aggregation rates of amyloidogenic polypeptide chains. *J. Mol. Biol.* **341**, 1317–1326 (2004).

43. A. P. Pawar, K. F. DuBay, J. Zurdo, F. Chiti, M. Vendruscolo, C. M. Dobson, Prediction of "aggregation-prone" and "aggregation-susceptible" regions in proteins associated with neurodegenerative diseases. *J. Mol. Biol.* **350**, 379–392 (2005).

44. G. G. Tartaglia, A. P. Pawar, S. Campioni, C. M. Dobson, F. Chiti, M. Vendruscolo, Prediction of aggregation-prone regions in structured proteins. *J. Mol. Biol.* **380**, 425–436 (2008).

45. G. G. Tartaglia, M. Vendruscolo, The Zyggregator method for predicting protein aggregation propensities. *Chem. Soc. Rev.* **37**, 1395–1401 (2008).

46. S. O. Garbuzynskiy, M. Y. Lobanov, O. V. Galzitskaya, FoldAmyloid: A method of prediction of amyloidogenic regions from protein sequence. *Bioinformatics* **26**, 326–332 (2010).

47. D. T. Seroski, X. Dong, K. M. Wong, R. Liu, Q. Shao, A. K. Paravastu, C. K. Hall, G. A. Hudalla, Charge guides pathway selection in β-sheet fibrillizing peptide co-assembly. *Commun. Chem.* **3**, 172 (2020).

48. E. Kangas, B. Tidor, Optimizing electrostatic affinity in ligand-receptor binding: Theory, computation, and ligand properties. *J. Chem. Phys.* **109**, 7522–7545 (1998).

49. E. Kangas, B. Tidor, Electrostatic complementarity at ligand binding sites: Application to chorismate mutase. *J. Phys. Chem. B* **105**, 880–888 (2001).

50. K. Zhu, M. R. Shirts, R. A. Friesner, Improved methods for side chain and loop predictions via the protein local optimization program: Variable dielectric model for implicitly improving the treatment of polarization effects. *J. Chem. Theory Comput.* **3**, 2108–2119 (2007).

51. J. Li, R. Abel, K. Zhu, Y. Cao, S. Zhao, R. A. Friesner, The VSGB 2.0 model: A next generation energy model for high resolution protein structure modeling. *Proteins* **79**, 2794–2812 (2011).

52. D. F. Green, A statistical framework for hierarchical methods in molecular simulation and design. *J. Chem. Theory Comput.* **6**, 1682–1697 (2010).

53. J. L. Fauchère, V. Pliška, Hydrophobic parameters Π of amino-acid side chains from the partitioning of N-acetyl-amino-acid amides. *Eur. J. Med. Chem.* **18**, 369–375 (1983).

54. S. D. Pickett, M. J. E. Sternberg, Empirical scale of side-chain conformational entropy in protein folding. *J. Mol. Biol.* **231**, 825–839 (1993).

55. P. Duan, K. Schmidt-Rohr, Composite-pulse and partially dipolar dephased multiCP for improved quantitative solid-state ^{13}C NMR. *J. Magn. Reason.* **285**, 68–78 (2017).
